# De-climatizing food security: Lessons from climate change micro-simulations in Peru

**DOI:** 10.1371/journal.pone.0222483

**Published:** 2019-09-27

**Authors:** Gustavo Anríquez, Gabriela Toledo

**Affiliations:** 1 Pontificia Universidad Católica de Chile, Departamento de Economía Agraria, Facultad de Agronomía e Ingeniería Forestal, Santiago, Chile; 2 Center for the Socioeconomic Impact of Environmental Policies, Santiago, Chile; Ball State University, UNITED STATES

## Abstract

This paper brings advances in weather data collection and modeling, and developments in socioeconomic climate microsimulations to bear on the analysis of the implications of climate change (CC) in the design of public policies to combat food insecurity. It uses new downscaled predictions of future climate in 2050, derived from three Earth System Models calibrated with a new historical weather station dataset for Peru. This climate data is used in a three-stage socioeconomic microsimulation model that includes climate risk, and deals with the endogeneity of incomes and simultaneity of expected food consumption and its variability. We estimate the impact of CC on agricultural yields, and find results consistent and fully bounded within what the global simulations literature has found, with yields falling up to 13% in some regions. However, we show that these drops (and increases) in yields translate to much smaller changes in food consumption, and also surprisingly, to very minor impacts on vulnerability to food insecurity. The document explores what explains this surprising result, showing that in addition to characteristics that are specific to Peru, there are household and market mediating mechanisms that are available in all countries, which explain how changes in yields, and corresponding farm incomes have a reduced impact in vulnerability to food insecurity. Finally, in light of these findings, we explore which policies might have greater impact in reducing food insecurity in contexts of hunger prevalence.

## 1. Introduction

In face of the growing and overwhelming evidence of large changes in global weather patterns, science from different disciplines has been called to provide evidence of the impacts of these global changes. A particular focus of attention for both scientist and funding institutions has been agri-food systems that are believed to be most acutely impacted by these climate changes (see for example, [1–3]). The present document is part of this fast-growing multidisciplinary effort: it brings advances in weather data collection and modeling, and developments in socioeconomic climate microsimulations to discuss the implications of climate change in the design of public policies that combat food insecurity.

Most of the literature that studies the climate change (CC) food security (FS) links reports the efforts of large-scale modeling [4–7]. These models generally predict future weather with General Circulation Models, which impact agricultural yields through Global Gridded Crop Models, which in turn affect Global Economic Models that make predictions about income and food consumption [8]. A good summary of what we learn from these models is available in [9]. However, a few highlights are important to stress here. Yields are expected to rise (particularly when carbon fertilization is included) in some global regions and fall in others, but the overall global net (average) effect appears to be negative, as a result of the marginal effect of CC. This is in spite of the fact that overall towards 2050, both area and yields are expected to continue their historical trends of growth. Models tend to agree that the most serious impacts on FS are expected to occur in low latitude areas of Sub-Saharan Africa and South Asia. Crop-specific yields are predicted to fall as much as 30% [10], as a result of CC, however, after allowing for these models to predict reallocation of area and crops, the overall net (marginal) impact on yields is more centered around (negative) 7–10% [11].

These type of models have been criticized in the literature because beyond crop and area selection, these models ignore adaptive and mitigating behavior [12], so perhaps they should be understood as a ceiling for the negative impacts. For example, CC impacts would be lower if one considers that agronomic adaptation is expected to increase yields by 15–18% [9]. We also learn from these models that it would be a mistake to stop at the yields effects to predict impacts on human welfare, because there are large predicted responses (adaptation) in production and trade as a response to climate shocks [8]; an argument that is furthered in this paper. In this respect, the large-scale gridded models proposed by [13] represent an important improvement in the right direction, by using a Ricardian land-value based approach [14], which assumes farmers adapt their best production strategy according to climate change. These models predict a more moderate impact of CC as compared to global gridded models.

On the other hand, we learn from the Global Gridded Crop Models, that the weather data derived from global circulation models (and other similar approaches) produce very poor predictions that are highly divergent from that obtained from control real weather data [15], questioning the reliability of the climate impacts in these models. The other criticism widely posed against these global models relates to its scale of analysis, they are unable to disentangle what are believed to be important sub-national variations in the impacts [4,7]. Hence, these models cannot inform local policymaking in terms of identifying the types of households most at risk, or the most successful adaptation practices on the ground. This is the type of microanalysis carried by another (smaller) strand of the literature to which this paper contributes.

Micro studies, link with varying degrees of sophistication the impacts of climate on welfare by fitting microeconomic models using household surveys and weather or climate information, and mimic the impacts of climate change by simulating shocks on weather variables. In Nicaragua, for example, a low-latitude country, additional warming of average temperatures has a significant negative impact on household agricultural income, as well as food security vulnerability [16]. In rural Mexico, [17] found that households, particularly in semi-arid regions were vulnerable to negative precipitation shocks; and also, in a finding related to what is argued in this paper, found that across regions, those with less assets were more vulnerable to weather shocks. [18] study in Uganda the impact of weather shocks on welfare indicators, like food consumption, and found very small to no impacts at all, attributing the result to consumption smoothing by households. CC adaptation strategies are studied by [19] in Uganda. They report that CC may hinder the viability of more modern and productive strategies (i.e. use of high yielding varieties (HYV) with increased fertilizer use), which is consistent with what was found in Ethiopia by [20].

The study presented in this paper is a step forward in improving ground-level micro simulation of CC. First, instead of simulating temperature increases or declines in precipitation, it uses a new climate dataset, which provides climate projections towards 2050, in a higher resolution 10km grid for Peru. Obviously, this is a great improvement, because climate change is complex, and in a large and agroecologically diverse country like Peru, changes in temperatures and precipitation are expected to vary even in nearby areas. The micro-modeling presented in this work, also represents a step forward in the modeling of climate impacts on FS, following the work of [16,21] we develop a three-stage model that includes climate risk, and deals with endogeneity of incomes and simultaneity of expected consumption and its variability.

On the other hand, Peru is a very interesting country to study the impacts of CC on FS, in spite of the fact that it is not a poor country, but rather a low-middle-income country. Peru has notorious social disparities, with a large portion of its population, most of it living in the Andean region, suffering poverty, extreme poverty, and hunger. In addition, the country has many agroecological zones, marked by the presence of an arid coast, the Andes, and the Amazonian rainforest. In this context, we expect *a priori* heterogeneous impacts of CC that will depend on socio-economic and agroecological contexts.

In the work presented below, we estimate the impact of CC on agricultural yields, and find results consistent and fully bounded within what the literature that performs global simulations has found (i.e. [10]), with yields falling up to 20% in some regions. However, we show that these drops (and increases) in yields translate to much smaller changes in food consumption, and also surprisingly, to very minor impacts on vulnerability to food insecurity. The document explores what explains this surprising result, showing that characteristics that are specific to Peru, but also household and market mediating mechanisms more generally, explain how changes in yields, and corresponding farm incomes have a reduced impact in vulnerability to food insecurity.

The paper follows by presenting the methodology used to evaluate the impacts of CC on FS, followed by a description of all data sources used. The fourth section of the paper presents a brief summary of the results of the complete microsimulation model. Following the robust results that climate driven changes in yields have a minor impact on vulnerability to food insecurity, the fifth section of this paper unravels the characteristics of Peru and the transmission mechanisms that explain these low impacts; and; explore which policies could have a greater impact in reducing food insecurity in contexts of hunger prevalence. The final section concludes.

## 2. Methodology to calculate the vulnerability to undernourishment due to climate change

In this work we address the potential impact of CC on social welfare by modeling and measuring how changes in weather patterns alter agricultural productivity and how these impacts ultimately affect food security. We study the impact of climate change through agriculture both for the importance the sector has on the economies of rural households, 75% of which declare agricultural incomes, and because it is expected to be one of the economic activities most affected by CC. The impact of CC on agriculture will be strongly related to the characteristics of each socioeconomic system, as well as the mitigation and adaptation capacity those systems [22]. For example, a reduction in the amount of precipitation or a change in its distribution will likely affect more those households which are more dependent on rainfed agriculture as opposed to those who have access to irrigation infrastructure.

To measure the impacts of CC on wellbeing, we propose a measurement that adequately reflects the consequences on food security due to the high prevalence of hunger in rural Peru. Improved estimates prepared for this study show that 32% of the rural population in 2012 suffered from food shortage or caloric deficit. A possible indicator is the prevalence of caloric deficit (also referred to as undernourishment), the percentage of people that are below the minimum caloric requirements established for each sex/age group according to anthropometry. The caloric deficit measured at a certain point in time reveals the nutritional state in a static form, which can be influenced by negative shocks, like a bad harvest or seasonal unemployment, or positive shocks as well. The changes in climate, on the other hand, manifest themselves over the mid and long term, for which we would want an indicator of wellbeing that better reflects the reality of the household over the long-term. We choose the vulnerability to undernourishment as a dependent variable, as this measurement eliminates the positive and negative shocks of the households, to deliver the expected wellbeing of the household over the long term, and captures the capacity of the household to resist and confront adverse circumstances, such as CC. Also, vulnerability better reflects the dynamic nature of food security, unlike poverty food security is a dynamic concept, as its definition indicates: having enough food at all times. Following [21], vulnerability of the household to undernourishment *V*_*h*_ is defined as the probability (Pr) that the household will experience a caloric deficit in the future, that is to say, the probability that the caloric consumption *C*_*h*_ will be below the minimum required caloric consumption threshold *κ*_*h*_, of that household *h*, given its anthropometric characteristics and demographic composition.

$$V_{h}=\text{Pr}\left[\left(\text{ln}\;\kappa_{h}-lnC_{h}\right)>0\right]$$ (1)

**Fig 1:**
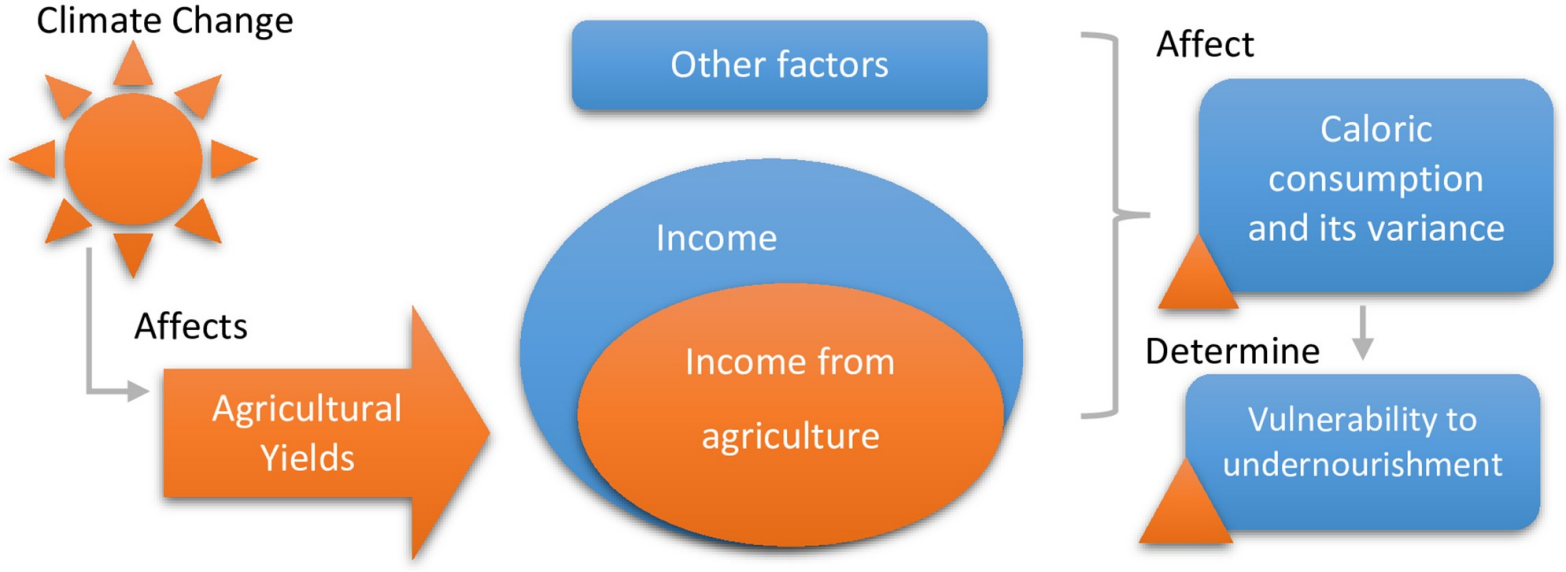
Conceptual model of the impact of climate change on vulnerability to undernourishment.

CC affects agriculture through changes in patterns of precipitation, minimum and maximum temperatures, and the temporal or seasonal distribution of these meteorological variables. The changes in these parameters will have repercussions on crop yields, which are transmitted directly to the farm (agricultural) income of the household (including own-consumption). Moreover, the caloric consumption *C*_*h*_ depends, among other factors, on the income of the household—agricultural and non-agricultural, as is observed in Fig 1, which has a preponderant role in the household’s access to food, be it bought or from their own production.

Based on this conceptual relation between climate change and vulnerability, we designed an econometric strategy to estimate the impact. The calculation of the probability of being in a condition of undernourishment implies information about the probability distribution function of caloric consumption of each household. In its projections of undernourishment, FAO utilizes a lognormal distribution for caloric consumption [23], which has also been empirically validated, for example [24]. Assuming the said distribution of caloric consumption, the vulnerability of each household is given by:

$$V_{h}=\overset{\text{ln}\;\kappa_{h}}{\underset{0}{\int }}\frac{1}{\sqrt{2\pi {\hat{\sigma }}_{h}^{2}}}\text{exp}\left\{-\frac{{\left(\text{ln}\;C_{h}-\text{ln}\;{\hat{C}}_{h}\right)}^{2}}{2{\hat{\sigma }}_{h}^{2}}\right\}d\text{ln}C_{h}$$ (2)

**Fig 2:**
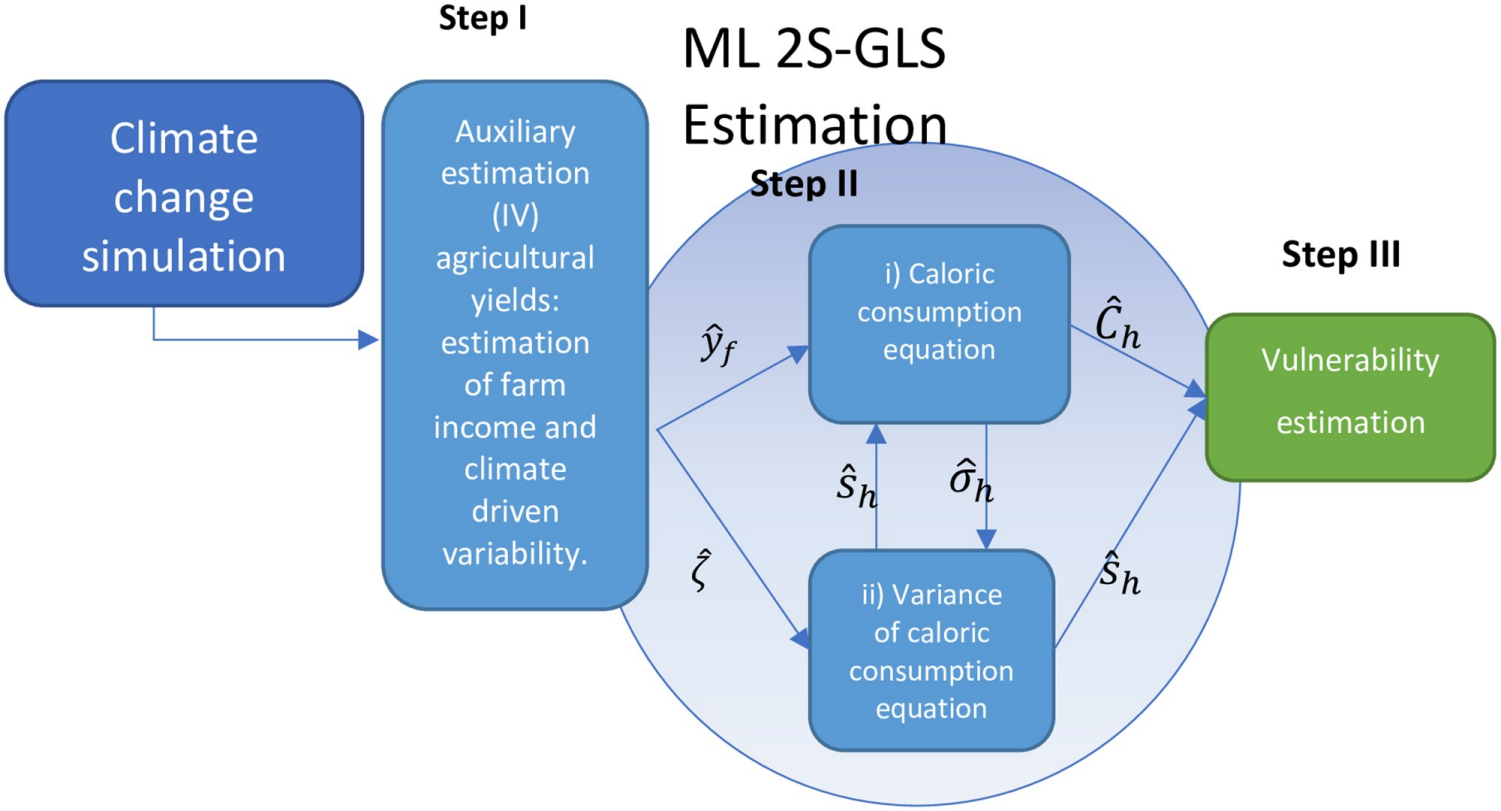
Model estimation of the impact of climate change on food insecurity of households.

To estimate the caloric consumption of household *C*_*h*_, an explanatory model (in reduced form) is presented whose main determinants are agricultural income *y*_*f*_ (the production of the household valued at market prices), and the non-agricultural income *y*_*nf*_. This model faces the challenges of isolating the decision of agricultural production from the decision of consumption, *e*.*g*. navigating the evident econometric problem of endogeneity between agricultural income and caloric consumption and addressing the heteroscedasticity present in caloric consumption, as by definition we know that less (more) vulnerable households present a lower (higher) variability in their consumption. To face these different challenges the estimation of vulnerability of each household is carried out in three main steps as illustrated in Fig 2. First, yields are estimated as a function of climate and inputs; from yields we estimate farm income and weather driven variability of yields which are used to estimate consumption and its variance in the second step, and; in a third step estimated consumption and its variance is used to estimate household-level vulnerability.

In the first step, agricultural incomes (*y*_*f*_) are estimated and predicted through an econometric model that explains agricultural yields. Step (I) is implicitly the first stage of an instrumental variables (IV) approach to the estimation of consumption in the second step. The IV model deals with endogeneity between caloric consumption and agricultural income. The model includes, among other things, agricultural inputs, characteristics of the producer, geographic characteristics, and climatic variables. Climatic variables are the primary instruments in predicting the agricultural income that is used in the second step. They represent good instruments as climate is highly correlated to yields (and therefore agricultural income), but it is not strongly correlated with consumption (criteria of exclusion). In addition to solving the problem of endogeneity between consumption and agricultural income, this first model delivers the mechanism by which to simulate CC impacts on the caloric consumption of the household. The predicted agricultural income (${\hat{y}}_{f}$) can be treated as exogenous and allows us to estimate the impact of agricultural income, and indirectly, of the climate, on caloric consumption. This step also predicts climatic risk ($\hat{\zeta }$), which is approximated by the variability of agricultural yields that is due to climatic variables. As explained below with the case of consumption, household-level yield variance can be estimated from the difference between observed and predicted yields. To estimate climate-driven risk, we regress household-level yield variance against climate variables, and use this predicted variance as a measure of climate-driven yield risk.

In the second step expected household food consumption and its variability is estimated. In this case, heteroscedasticity of consumption is expected, *i*.*e*. variance is not constant, but rather it reveals the level of vulnerability of a household due to the variability of expected consumption, which instead of being constant, as traditional econometrics assumes, is specific to the household. In the second step (II) we approach said heteroscedasticity utilizing Just and Pope’s model, which estimates both equations i) Caloric consumption equation and ii) Variance of caloric consumption equation in its log-linear form by generalized least squares, GLS (see Fig 2). In this work, we estimate both models jointly by maximum likelihood, which is equivalent to iterating Just and Pope’s recursive model until the regression parameters converge, see details in [25]. It is important to stress that this methodology not only deals with the problem of heteroscedasticity, but also deals with the perhaps a more relevant problem, the endogeneity of the variance of expected consumption.

In a third step (III) the prior results, the expected (i.e. predicted by the above model) household caloric consumption ${\hat{C}}_{h}$ and the variance of caloric consumption ${{\hat{S}}_{h}}^{2}$, are used to calculate the vulnerability indicator of each household *V*_*h*_, as shown in Eq (2). The simulations of climate change are derived from global climatic models with national data, see details below. With the simulated climate data, the predictions for variables of interest in steps (I) and (II) from the prior models are recalculated to have new estimates for the caloric deficit as well as the food vulnerability. In this way the methodology allows for the mapping of current food situation and vulnerability to undernourishment, versus the scenario with climate change. Additionally, it allows us to contrast the impact of climate change with the structural conditions that determine vulnerability to undernourishment.

## 3. Data sources

The objective of this multidisciplinary study requires the compatible use of distinct information sources, as is described here. The data used comes from four principal sources: 1) The National Household Survey (ENAHO, INEI) (four rounds, between 2005 and 2012); 2) Special anthropometric module (ENAHO 2012, INEI); 3) Historic climate data; multiple meteorological seasons (SENAMHI, National Service of Meteorology and Hydrology), and; 4) Future climate scenarios from three different Global Climate Model (ESMs) projections, downscaled to Peru in 10km grids.

The ENAHO (National Household Survey) allows us to satisfy the information requirements related to the measurement of: i) agricultural productivity, ii) food security, iii) factors that determine vulnerability to food insecurity of rural households. Information is obtained from the survey modules related to agricultural and livestock production (and related incomes): agricultural, livestock, and forest production, the input costs, and related variables (number, size, and use of parcels, tenure, and irrigation system, for example). Based on this information, we construct the key variables for the agricultural productivity model: production, yields, prices, and value of production indexes, using Törnqvist indexes. The reference period for agricultural income is the last 12 months. Additionally, we use available information about the socioeconomic, demographic, and cultural profiles of the rural household as explanatory factors of vulnerability to food insecurity.

Agricultural income had outliers removed, a procedure that consisted in analyzing income per hectare by socioeconomic groups and sampling stratum with an algorithm that removes extreme values that exceed three standard deviations from the median. A standard procedure in the literature, cf. (Davis et al., 2010). The data is a cross-section from the period 2005 to 2012—to take into account the most recent developments, the data available from the ENAHO are from 2005, 2007, 2010, and 2012, as Table 1 shows. Finally, all of the monetary values are expressed in 2012 prices. The deflation is carried out using the CPI for Metropolitan Lima. The survey is representative at the national level, for rural and urban zones and the 24 departments (regions) of the country.

**Table 1:** National Household Survey (ENAHO) coverage for urban, rural, and agricultural households.

ENAHO Results	2005	2007	2010	2012
**Total Households**	19,895	22,204	21,496	25,091
**Urban households**	11,080	13,560	12,962	15,355
**Rural households**	8,815	8,644	8,534	9,736
**Agricultural households**	8,781	9,103	9,194	10,389

Source: Authors’ calculations

To measure the level of food insecurity, the caloric consumption per person was calculated using the detailed listing of food consumption inside and outside the home, which is converted to calories using the CENAN (National Center for Food and Nutrition) Peruvian food conversion tables [26].

ENAHO’s 2012 anthropometric measurements module was used to identify the height distribution by age in Peru. With this information the minimum dietary energy requirements (MDER) were calculated by gender for each age group in Peru, methods in [27,28]. The contrast between the required minimums and the caloric consumption, both measured at the household level, lead to one food security indicator—caloric deficit.

### 3.1 Climate modeling and simulations

The climate data used here is a new dataset of climate projections for Peru, derived from the downscaling [29] of global climate projections of three Earth System Models (ESMs) from the Fifth Assessment of the IPCC [1]. The statistical downscaling of climate projections for different Green House Gases (GHG) emissions scenarios was carried out by the National Meteorology and Hydrology Service of Peru (SENAMHI) for the purpose of this study [30]. Three ESMs used are CanESM2 (Canadian Earth System Model), CNRM-CM5 (National Meteorological Research Center of France) and MPI-ESM-MR (Max Planck Institute for Meteorology). The global temperature and precipitation projections on coarse grids through the year 2099 at the daily scale under two GHG (greenhouse gases) concentration scenarios (Representative Concentration Pathways—RCP4.5 and 8.5) were statistically downscaled to weather station locations in Peru after calibration with historical meteorological data (1971–2010). The calibration of statistical methods used 105 stations for maximum temperatures, 102 stations for minimum temperatures, and 265 stations for precipitation. These stations were selected after taking into account a percentage of lost, missing, or non-collected data in the period 1971–2000 and 1981–2010 below 20%.

The downscaled climate projections on a daily scale were further temporally aggregated on a monthly timescale, and then spatially interpolated by considering the proximity to the ocean, and other geographic influences on climate to a spatial resolution of 10 x 10 km. The information from the spatial interpolations was aggregated at the administrative unit (department and province). To link the climatological information with the household information, average values at the province level were ultimately used. In the simulations of vulnerability with climate change, average values for the 5 years centered around 2050 (2048–2052) were used to capture climate trends and not inter-annual climate variability. Therefore, the correct interpretation of the simulations is: What would the impact on food vulnerability be in Peru if it faces today the climate that we expect to see towards 2050? In the exposition below, we present the tables of modeling and simulations derived from the CanESM2 climate model and for the RCP 4.5 and 8.5 scenarios of moderate and high GHG emissions growth. However, all six models and their related tables are available in supplementary tables (see S9, S10, S11, S12, S13, S14 and S15 Tables), and; also below, we present mean and inter-model variability, across the six climate scenarios, for the main results discussed. Further, climate estimates towards 2050 for all three ESMs are also available in the S6, S7 and S8 Tables.

## 4. Results

### 4.1 Estimation of agricultural yields

Agricultural productivity is estimated through a model that represents a Cobb-Douglas production function expanded to control for characteristics of the household, the farmer, geography, and climate. In an abbreviated form the model is:

Y=F(I,K,FC,HC,G,CI,FE).

Where,

Y: Yields (index of production per hectare)

I: Agricultural inputs

K: Physical Capital

FC: Farmer characteristics

HC: Household characteristics

G: Geographic characteristics

CI: Weather variables

FE: Fixed effects (time and region)

The dependent variable *Y* provides the crop yields per hectare. When analyzing agricultural productivity, we face the challenge of appropriately aggregating in an index the hundreds of crops that exist in a country with the agro-climatic diversity of Peru. On the other hand, it is necessary to consider that the choice of each crop is endogenous, which is to stay, determined in the biophysical context, of prices and preferences, which implies that to analyze each crop separately could distort the results of the impact of CC as the farmer optimizes the input assignments in an optimum mix of distinct crops. We offer a solution to these problems through the construction of a Törnqvist [31] production quantity index. This index was chosen (and others rejected) as in its construction it considers each crop according to the product’s share in the total value of production of a representative producer (we use the average of the quantities between producers as the representative producer), but also considering the share of the same crop in the total production of each farmer. Thus, a better index is constructed in contexts such as the case of a particular crop that could be the only source of income for a particular farmer, but irrelevant on the national scale. The index is also comparable between years as prices were deflated, and the representative farmer is calculated between farmers and years that the study covers. Table 2 shows the evolution of estimated output (e.g. the Törnqvist quantity index).

**Table 2:** Evolution of agricultural output (Törnqvist index).

Year	Average	SD	N
2005	1.50	0.82	7,622
2007	1.61	0.87	7,871
2010	1.78	0.93	8,003
2012	1.76	0.94	8,970
Total	1.66	0.90	32,466

Source: Authors’ calculations using ENAHO.

While there is a long tradition in the agricultural economics literature of using household surveys in econometric models that explain yields, since at least [32], there is much less literature using climate variables within this type of models. Hence, climate specification model selection is based in minimizing the Bayesian Information Criterion (BIC), that punishes more heavily parameter addition as compared to the also popular Akaike Information Criterion. A table with a selection of climate model specifications considered, and how they fared with different statistics with respect to the model presented here is included in S1 Table. Including climate in these yield models presumes the tackling several challenges, among them: *i*) given the agro-climatic heterogeneity of Peru, distinguishing climatic zones for area-specific weather variable impacts, *ii*) deciding the cutoff month for the agricultural year, *iii*) including variables that reflect the climatic variability without producing autocorrelation of errors.

The agro-climatic heterogeneity of Peru implies that the increase in frequency of an event (for example precipitation) does not have the same impact in the different regions of Peru, i.e. rain in the arid coast or the tropical rainforests. We considered climate variables having differentiated impacts by eco-regions (coast, mountains, rainforests / selva) and by survey strata (i.e. North Coast, Center Coast, etc.), but chose the former based on the BIC. Furthermore, climate variables need to reflect 12 months of data, as the production information from the household survey is annual. Ideally, we would like these 12 months to reflect the agricultural year according to harvests, instead of the calendar year. Nevertheless, the agricultural calendar varies by agro-climatic zone. Distinct alternatives such as different cut-off months by administrative region, and eco-region were explored, but based on the goodness of fit a country-wide agricultural year ending on July was chosen.

The climate projections dataset consists of monthly information on average minimum and maximum temperatures and total precipitation, by department or province. The choice of which climatic attributes to include, given this data, requires giving flexibility to the estimation by including different climate measurements, but at the same time minimizing the potential for autocorrelation of errors and multicollinearity. Additionally, the special nature of cross-sectional and time series data was considered. The dataset allows the use of average climate conditions over time, which would reflect impact of longer-term climate conditions; and each year’s meteorological conditions, which would capture the response of yields to the specific year’s conditions. We use cumulative moving averages of each climate indicator (i.e. the average from 1970 to the year of the observation); and the annual deviation, that is the indicator, say total precipitation, for that year *t* and province *i* minus the cumulative average of total annual precipitation from 1970 to year *t*, for province *i*: $\left(x_{it}-{\overset{-}{x}}_{it}\right)$. Averages do not capture the concentration or dispersion of climatic indicator within a year, which is of particular importance with precipitation, so in addition to total precipitation, we use indicators of intra annual dispersion (the seasonality index of [33]). Given the original climate data, we settle for four underlying indicators, maximum temperatures, average temperatures (using min. and max. data), total precipitation, and precipitation seasonality. This information is expanded to a vector of 24 climate variables: each climate indicator as a cumulative moving average, and as an annual deviation, and expanded to the three main eco-regions. Also, the impact on temperature on yields may vary with altitude, so in addition to controlling for altitude we explored the interaction of altitude with the temperature indicators. Based on the BIC we limited this interaction to mean temperatures and only in the mountains / sierra ecoregion. A sensible outcome, given that large altitude differentials are observed in this ecoregion and not on others.

The estimation of this agricultural productivity model is done through ordinary least squares, and includes agricultural producers: 32,466 observations with complete information. The impact of distinct groups of variables on productivity is briefly discussed. The agricultural inputs, as expected, are key to crop yields, principally labor, variable inputs and physical capital; and, as expected diminishing returns to scale are estimated. All the continuous variables of agricultural inputs of farming households are expressed per-unit (ha) of agricultural (operated) land to indicate the intensity in the use of inputs. Spending on hired labor is positive and significant, but not the number of household workers. Additionally, agricultural spending is positive and significant, signaling the importance of variables inputs: fertilizers, and other agrochemical products. Physical capital is approximated from variables such as, animals, motorized vehicles and an (local area) infrastructure index. Both the infrastructure and household assets index, are principal components indices. The infrastructure index is composed of indicators of connectivity and proximity to services, like distance to health center, telephone and electricity connection, and public trash collection services. The presence of motorized vehicles and the infrastructure index presents a positive effect on the yield per hectare, however no type of animal appears to be related to agricultural productivity.

In addition to the input variables normally included in a Cobb-Douglas function, in this estimation characteristics of the farmers and households, as well as geographic variables and location specific fixed-effects, are controlled for. The majority of relationships found are within what was expected, highlighting the farmer’s dedication (share of agricultural income in total income), the age that shows a quadratic relationship, and the altitude of the property, negatively related to yields.

There are several ways to gauge the contribution of the climate variables to this yields model. Here we focus in the contribution of the climate vector in explaining yield variability, and the marginal contribution of each climate attribute to yields by eco-region. The model explains 72% of yield variability, the R-squared. This statistic cannot be linearly decomposed, because the order in which variables are entered to the model affect its contribution, which is why researchers usually average the contribution of variables over all possible orderings, the Lindeman, Merenda and Gold or Shapley decomposition, see [34]. The first column of Table 3 shows in parenthesis the contribution (in percentage) of each variable group to the total R-squared. Not surprisingly, inputs (land, seeds, fertilizer, employment, etc.) is the most important set of variables, accounting for 70% of explained variability. Climate accounts for 10% of explained variability, and provides an equivalent contribution to all geographic, time and location fixed effects variables (31 variables in total). Another way to assess the contribution of the climate vector to the yields model is to look at the marginal contribution of each climate attribute to yields. The results presented in Table 3 show that climate vector is not only highly significant in explaining observed yields, but provides sensible results. The complete climate vector, as well as each climate indicator (high and average temperatures, precipitation, and seasonality) are all significant with *p*-val. < 0.001. It is not easy to see from Table 3, the marginal impact of each climate attribute on yields. It can be shown that the marginal impact of, say maximum temperature in the Coast, would be: $\frac{\beta [Max.\;temp.\;average-Coast]}{T}+\frac{\left(T-1)\beta [Max.\;temp.\;dev.-Coast\right]}{T}$, where *T* is 38, the years from 1970 to 2008 the year around which the data is centered. These marginal impacts by variable and eco-region are summarized in Table 4. In the case of the Coast region, where agriculture is almost exclusively irrigated (being extremely arid, off-river-valleys agriculture is practically non-existent), precipitation impacts yields marginally, but note that precipitation in the coast is negatively correlated with precipitation in the Mountains, which is ultimately relevant for irrigation and yields in the coast (see correlation matrix in S2 Table). Higher temperatures are correlated with higher yields, given that the hotter Northern Coast is more productive, while higher mean temperatures are consistent with lower yields, likely due to heat stress. On the mountains, where there is both irrigated and rainfed agriculture, province-level atmospheric temperature is not significant, because temperature changes more drastically with altitude rather than climate. Rain is an important driver of yields, with yields responding positively to well-marked rainy seasons, while years with excessive rain and spread throughout the year negatively impacts yields.

**Table 3:** Estimation results of agricultural productivity (yields).

Dependent Variable ln (index of agricultural production/hectares of operated land)		Coefficient	*t*-stat
I: Agricultural Inputs (69.8)	ln hectares of operated land	-0.677***	35.99
	ln number of hh agricultural workers	-0.00158	0.0905
	ln spending on agricultural labor	0.0385***	14.92
	ln spending on agricultural variable inputs	0.202***	31.17
	ln spending on livestock variable inputs	0.0158***	4.352
	Irrigation dummies: drip, gravity-fed, wells	yes	
K: Physical Capital (3.9)	Animal dummies: horses, cows and llamas	yes	
	ln infrastructure index	0.101***	9.082
	Motorcycle dummy	0.0619**	2.42
	Car or truck dummy	0.155***	5.294
FC: Farmer Characteristics (1)	Male head of household dummy	0.0996***	6.083
	ln years of schooling of head of household	0.0257***	4.034
	ln age of head of household	1.313***	3.69
	ln age of head of household squared	-0.179***	3.856
	Head of household speaks indigenous language dummy	-0.0796***	3.397
HC: Household Chars. (1.8)	ln number of people in the household	0.224***	10.64
	Percentage of people in the household who do not work	-0.201***	5.154
	Share of agricultural income in total income	0.0119***	36.14
Cl: Climate Variables (10.3)	Maximum temperature (cumulative moving average)—Coast	-0.107	1.539
	Maximum temperature (cumulative moving average)—Mountains	-0.124***	3.141
	Maximum temperature (cumulative moving average)—Rainforest	-0.0666	0.743
	Maximum temperature deviation period—Coast	0.354***	2.894
	Maximum temperature deviation period—Mountains	-0.0477	0.759
	Maximum temperature deviation period—Rainforest	-0.573***	5.393
	Average temperature (cumulative moving average)—Coast	0.162***	2.704
	Average temperature (cumulative moving average)—Mountains	0.139***	3.858
	Average temperature (cumulative moving average)—Rainforest	0.0835	1.081
	Average temperature deviation period—Coast	-0.320**	2.558
	Average temperature deviation period—Mountains	0.210*	1.816
	Average temperature deviation period—Rainforest	0.820***	9.037
	Precipitation (cumulative moving average)—Coast	-0.000202	0.704
	Precipitation (cumulative moving average)—Mountains	0.000278***	2.736
	Precipitation (cumulative moving average)—Rainforest	0.000158***	2.739
	Precipitation deviation period—Coast	0.000336*	1.713
	Precipitation deviation period—Mountains	-0.000764***	7.549
	Precipitation deviation period—Rainforest	-0.000209**	2.273
	Index of seasonal precipitation (cumulative moving average)—Coast	-0.149	0.479
	Index of seasonal precipitation (cumul. moving average)—Mountains	0.123	0.435
	Index of seasonal precipitation (cumul. moving average)—Rainforest	2.779***	3.719
	Deviation period of index of seasonal precipitation—Coast	0.0911	0.463
	Deviation period of index of seasonal precipitation—Mountains	0.568***	3.417
	Deviation period of index of seasonal precipitation—Rainforest	1.776***	5.732
	Average temperature (cumulative MA)–Mountains x Altitude	-1.13E-06	0.552
	Average temperature deviation period–Mountains x Altitude	-9.37e-05***	3.043
G, FE: Geographic chars. and fixed effects (13.1)	Altitude	-5.66e-05***	2.715
	Latitude*	0.0015	0.362
	Year dummies: 2005, 2007, 2010, 2012	yes	
	Eco-region dummies: coast, mountains, forests	yes	
	Department dummies	yes	
	Observations	32,466	
	R-squared	0.717	
	Initial Log-likelihood	-61027	
	Final Log-likelihood	-40545	
	AIC	81250	
	BIC	81921	

Notes: Robust *t* statistics in the fourth column.

*** *p*-value <0.01,

** *p*-value <0.05,

* *p*-value <0.1

The effect of longitude is implicitly captured by the three eco-region dummies. Values in parentheses in the first column indicate the percentage added by the group of variables to total R-squared according to the Shapley decomposition (see text). Also note that in this equation we use observed, historical climate data, which has been downscaled. Full regression available in S3 Table.

**Table 4:** Impact of climate variables by eco-region.

	Max. Temp		Mean Temp		Precipitation		Seasonality	
Coast	0.342	***	-0.307	**	0.0003	*	0.085	
Mountains	-0.050		-0.060		-0.0007	***	0.556	***
Rainforest	-0.556	***	0.800	***	-0.0002	**	1.802	***

Note: Calculated by authors with information from estimates presented in Table 3. Significance at 10%, 5%, and 1% marked with (*), (**), and (***), respectively.

Through the present model, the climatic variables affect the crops yields, which is how climate change influences household wellbeing. Agricultural yields can also be predicted under different scenarios of CC, using the predicted climate for different ESMs and GHG emission growth scenarios. Table 5 shows the percentage changes of agricultural yields under different future emission scenarios. It is shown, in column 5, that the yield index increases, on average, 1.6% for the RCP 4.5 scenario of moderate GHG emission growth (0.1% inter model mean, with a 4.5% standard deviation). However, these modest averages hide a pronounced heterogeneity, by geographic region the yields per hectare changes between -20% and 24.7%, the southern mountain and the southern coast being the geographic regions most affected. Additionally, it is possible to observe that the changes are sensitive to the intensity of CC, for example, the impact changes from positive to negative in all of the mountain regions (column 6 and 8).

**Table 5:** Effect of the climate change on yields, 2050 vs. 2012.

Region	Yield per hectare (index)			Climate simulations			
	No. hh	Base Line	Prediction	CanES 4.5	dif %	CanES 8.5	dif %
*N*. *coast*	105,954	6.645	6.586	6.688	10.2	6.752	16.6
*Central coast*	24,522	7.180	7.332	7.430	9.8	7.515	18.3
*S*. *Coast*	10,629	6.519	6.615	6.789	17.5	6.862	24.7
*N*. *sierra*	327,174	5.303	5.304	5.435	13.1	5.266	-3.8
*Center sierra*	461,774	5.840	5.840	5.868	2.8	5.748	-9.2
*S*. *sierra*	488,793	5.431	5.563	5.568	0.5	5.364	-19.9
*Rainforest*	453,161	5.179	5.150	5.023	-12.7	5.030	-11.9
Total	1,931,197	5.566	5.593	5.609	1.6	5.497	-9.6%

Source: Authors’ calculations

### 4.2 Consumption and volatility of consumption estimate

To estimate the caloric consumption of households, a model that simultaneously estimates caloric consumption and variance of said consumption is used. This model assumes endogeneity of farm income, as explained above. We apply a log-linear model based on the empirical observation that the consumption of dietary energy follows the lognormal distribution:

ln(Cal)=F(HHC,HC,CE,G,FE)

ln(VC)=F(HHC,HC,CE,G,FE)

with:

Cal: calories per capita per household

VC: variance of calories per capita per household

HHC: Characteristics of the head of household

HC: Characteristics of the household

CE: Characteristics of the locality where the household is located

G: Geography

FE: Fixed effects (time and region)

As described above, the model is estimated by maximum likelihood, which is equivalent to reiterating the recursive model of both, consumption and variance, in their log-linear form by GLS following the steps proposed by Just and Pope in their stochastic production model [25,35]. This design implies that climate change affects caloric consumption, both via agricultural income and climatic risk. The principal determinants of consumption are agricultural and non-agricultural income, and additionally characteristics of the household and head of household are included. To control the effects of non-observable time invariant characteristics we use geographic characteristics and fixed effects at the department level, as well as time fixed effects to control for country-wide temporal shocks.

It is expected that the caloric consumption is positively related to attributes that increase the access to food, like incomes and education, and negatively related to those that reduce it, like unemployment. With respect to the variance in caloric consumption, it is expected that it is positively related to attributes that increase the vulnerability of a household, like household economic dependency, household size, and also variables that increase caloric consumption, but are simultaneously sources of dispersion, like agricultural income which by its nature is more variable. In contrast, the variables that should be negatively related to variance of caloric consumption are those which diminish the vulnerability of the household, acting as support in situations of need, variables such as the assets of the household, the social capital (including marriage), and some public assistance policies.

First, observe in Table 6 that, being an agricultural income-earning household contributes to the increase in calories per capita consumed, and at the same time decreases their vulnerability. This finding allows one to understand that subsistence agriculture, from a nutritional perspective, plays a role beyond generation of income, contributing in a significant way to the food security of its members (it is also a likely signal of market failures, see below). But at the same time, the greater the percentage of household income coming from agriculture, the lower the caloric consumption; that is to say, those households highly dependent on agricultural activity are the most vulnerable. It is this group that faces the highest food security risks in the face of CC, given that their income depends to a greater extent on the success of agricultural activities. As expected, agricultural income as well as non-agricultural income increase caloric consumption, and at the same time, both increase the volatility of consumption (we find that the elasticity of consumption with respect to non-agricultural income is greater than agricultural income).

**Table 6:** Estimated results of determinants of caloric consumption and the variance of caloric consumption.

Name of variables	Caloric Consumption	Consumption Variance
Climatic Risk	-0.00434***	-0.0117***
	(4.723)	(4.050)
Dummy agricultural household	0.115***	-0.468***
	(9.662)	(13.440)
Dummy male head of household	0.00777	-0.122***
	(0.850)	(4.200)
ln schooling years of the head of household	0.00208***	-0.00541**
	(3.286)	(2.450)
ln age of the head of household	0.0123***	-0.0310***
	(11.200)	(10.120)
ln age of the head of household squared	-0.000134***	0.000326***
	(12.800)	(11.290)
dummy head of household married or cohabitating	-0.0115**	-0.0949***
	(2.084)	(4.982)
dummy head of household widowed	0.00506	0.108***
	(0.491)	(3.826)
dummy head of household speaks an indigenous tongue language	-0.00761	-0.00543
	(0.968)	(0.221)
Percent of people in the household who do not work	-0.231***	0.165***
	(20.800)	(5.323)
ln household size	-0.0813***	-0.0813***
	(43.420)	(14.270)
ln no. women in the household	-0.0977***	-0.314***
	(8.171)	(8.748)
Average schooling years of the household	-0.00226**	0.00858***
	(2.246)	(2.692)
Infrastructure index	0.0473***	-0.172***
	(10.220)	(11.270)
Assets index	0.0167***	-0.107***
	(2.974)	(5.432)
School dropout member dummy	-0.0415***	0.0737***
	(6.232)	(3.152)
Participates in Vaso de Leche program	-0.0256***	-0.132***
	(4.957)	(7.475)
Participates in soup kitchens	0.0784***	-0.158***
	(8.465)	(4.794)
Share of agricultural income in total income	-0.000155**	0.000178
	(2.396)	(0.842)
Index of value of agricultural production (agricultural Income—predicted)	0.0261***	0.0298***
	(14.670)	(8.330)
Non-agricultural income	2.68e-05***	1.99e-05***
	(21.470)	(8.536)
Sierra–Mountain Region	-0.104***	0.352***
	(8.390)	(9.249)
Selva–Rainforest Region	-0.0487***	0.0714
	(3.264)	(1.497)
Year 2007	0.0157**	-0.113***
	(2.344)	(5.060)
Year 2010	-0.0784***	0.00254
	(10.550)	(0.108)
Year 2012	-0.0288***	0.0479*
	(3.727)	(1.950)
Constant	7.860***	-0.0997
	(229.2)	(1.006)
Observations	35,358	35,358

Note: Significance at 10%, 5%, and 1% marked with (*), (**), and (***), respectively. Full regression available in S4 Table.

As expected, both the infrastructure index and the assets index are factors that unequivocally diminish the vulnerability of the household, they are associated with a higher caloric consumption and unlike income they are associated with a lower variance of caloric consumption as well. Besides indicating relative wealth, assets can act as an economic buffer for bad times, for example, being sold to buy food.

With respect to other relevant variables, the percentage of people in the household who do not work increase vulnerability. The number of adults in the household as well as the number of women diminish the caloric consumption per capita, but also diminishes the volatility of said consumption. Of the characteristics of the head of household, being a man, having a higher education level, reduce the vulnerability of the household, while the age shows an expected quadratic relationship, increases the consumption during youth, but reduces it during old age. Participation in the *Vaso de Leche* (glass of milk) program does not increase caloric consumption, probably because these are the households already vulnerable, but it does accomplish diminishing the volatility of caloric consumption, diminishing vulnerability. On the other hand, participating in soup kitchens unequivocally diminishes the vulnerability of the household. Note however, that this model cannot be interpreted as an evaluation of public policies. Here the participation in programs is used only as a control variable. To adequately evaluate the programs one must deal with the self-selection bias of these programs, which is not done here.

### 4.3 Impacts on vulnerability

In analyzing food security under the effect of climate change we are implicitly referring to the future. The concept of vulnerability best reflects the most chronic characteristics of food insecurity. While the caloric deficit accounts for the scarcity or abundance of food in a current situation, vulnerability accounts for the probability of being in a situation of undernourishment be it in the present or the future [21] and it is this variable that indicates the nutritional wellbeing of the household in the most stable form.

Empirically, the caloric deficit differs considerably from the vulnerability as can be observed in Table 7. The groups that present caloric deficit or vulnerability, but not both at the same time, represent 14.8% and 16.3% of the population respectively. While the proportion of vulnerable households and those that suffer caloric deficits are a similar quantity +/- 33% of the population, they are distinct groups as almost half of the vulnerable population did not experience caloric deficit in 2012.

**Table 7:** Relationship between caloric deficit and vulnerability of households in 2012.

		Vulnerability		
Caloric Deficit		Not vulnerable	Vulnerable	Total
Not calorically deficient	People	3,959,854	1,249,4446	5,209,301
	Percentage	51.76%	16.33%	68.10%
	Calories	3,085	2,742	3,002
Calorically deficient	People	1,134,552	1,305,995	2,440,546
	Percentage	14.83%	17.07%	31.90%
	Calories	1,406	1,368	1,386
Total	People	5,094,406	2,555,441	7,649,847
	Percentage	66.59%	33.41%	100%
	Calories	2,710	2,042	2,487

Source: Authors’ calculations

To measure the effect of climate on vulnerability the predicted climate from the climatic simulation models was used (see details above). The climate affects food consumption through agricultural yields, agricultural income, and production for own-consumption. The climatic model presented throughout this section is CanES in the two GHG emission scenarios, moderate climate change 4.5 and greater than expected climate change 8.5.

Here we focus on the vulnerability results, but it is important to note that the impacts on caloric deficits are more pronounced. In the base line, as shown in Table 8, the sierra (Andes region) presents the highest average probabilities of being undernourished (30–40%), in contrast with the coast, where the probability ranges between 22 and 25%. On the coast, the lower vulnerability is explained by the fact that the population is located closer to urban centers and enjoys higher public infrastructure development, among other factors that diminish vulnerability.

**Table 8:** Simulated changes in vulnerability, 2012.

Region	N° people	Base Line	Probability of vulnerability			
			CanES 4.5	Change 4.5	CanES 8.5	Change 8.5
*N*. *coast*	577,462	23.91%	23.68%	-0.96%	23.54%	-1.55%
*Central*. *coast*	201,227	23.30%	23.12%	-0.77%	22.99%	-1.33%
*S*. *coast*	69,564	24.85%	24.48%	-1.49%	24.34%	-2.05%
*N*. *sierra*	1,435,993	40.55%	40.41%	-0.35%	40.56%	0.02%
*Central sierra*	2,024,206	37.81%	37.75%	-0.16%	37.87%	0.16%
*S*. *sierra*	1,664,126	30.87%	30.83%	-0.13%	31.07%	0.65%
*Rainforest*	1,677,269	31.76%	32.00%	0.76%	31.95%	0.60%
Total	7,649,847	33.94%	33.91%	-0.09%	34.01%	0.21%

Source: Authors’ calculations.

Table 8 shows that the observed changes in vulnerability as a result of the climate simulations, both nationally, and at the regional levels are very small. Under the moderate 4.5 scenario countrywide vulnerability hardly changes on average, -0.09% (0.03% inter climate model mean, with 0.1% standard deviation). However, opening the average to geographic regions, a decrease in vulnerability is observed on the coast and the mountains—both zones experience an increase in agricultural yields under this model. Likewise, the selva / jungle region shows an increase in the observed vulnerability, a result in line with the fact that the rainforest is the only region where there is a predicted decrease in yields in the moderate emissions growth scenario.

For the 8.5 scenario of greater climate change, the vulnerability of the overall population increases by 0.2%, less than a percentage point. Said increase is small, but diverse between geographic regions, differentiating the coast which is the only region where vulnerability decreased, while it increased in the sierra and the rainforest.

The results of the impacts of CC on vulnerability are not univocal; the impacts depend on how the climate affects the crops typical to the area (see Table 5). However, the result that climate change has a very moderate effect on vulnerability to food insecurity is one of the most robust and relevant result presented in this study. This result of moderate impact is robust across climate models as can be checked in S14 and S15 Tables. Nevertheless, this result should not be interpreted as climate change not having a considerable impact on agricultural yields (and their corresponding incomes, as shown above), but rather that the transmission to food consumption of this impact is lessened by a series of factors that mediate the relationship between agricultural productivity and food security, as is discussed in the next section.

## 5. Climate change and vulnerability: The main lessons

In the following section we reconcile two seemingly contradictory results observed in this study: the considerable impact of CC on yields, but the moderate impact on vulnerability to food insecurity. This exercise involves identifying the factors that mediate the relationship between climate change and food security vulnerability. First, we discuss characteristics (biophysical and socioeconomic) that impact the results observed and are exclusive to Peru; and second, we identify characteristics that are specific to the way in which the impacts of CC are generally transmitted to vulnerability to hunger.

Peru possesses fundamental characteristics that strengthen its resilience when facing climate change: the agro-climatic heterogeneity and the diversification of income generating activities. First, the agro-climatic heterogeneity of Peru means that the changes in climatic variables do not imply a univocal relationship between climate and agricultural productivity. To the contrary, it implies the coexistence of gaining and losing eco-regions in terms of agricultural yields (see Table 5), these gains and losses countervail each other giving way to a moderate mean national impact, the same occurs at the department level of each eco-region. Additionally, this agro-climatic heterogeneity occurs within small geographic areas, which offers high potential for adaptation. In realizing the simulation, we assume that the farmers produce the same crops that they produced before, ignoring the potential for adaptation of their farming strategies. However, this assumption is not realistic, it is highly likely that an adaptive response to changes in climate exists for crops, either through intensified use of land for crops benefited by the climate or by changing the choice of crops produced, by altering the timing of agricultural activities, among other adaptive agricultural practices, [19].

Secondly, the diversification of income generating activities at the household level favors, at the same time, the capacity of the households to confront scarcity of agricultural income due to climate change. Although the economy of rural Peru has a strong agricultural component in comparison to other similarly developed economies (see comparisons in [36]), the percentage of household income from own-agricultural production is only 28%, and around 65% of the households’ incomes come from sources not related to agriculture. The development of a non-agricultural rural economy allows climatic and other shocks to be mitigated, so reductions in agricultural income can be dealt with a greater dedication to the rural non-farm economy, which is fairly developed in Peru.

**Fig 3:**
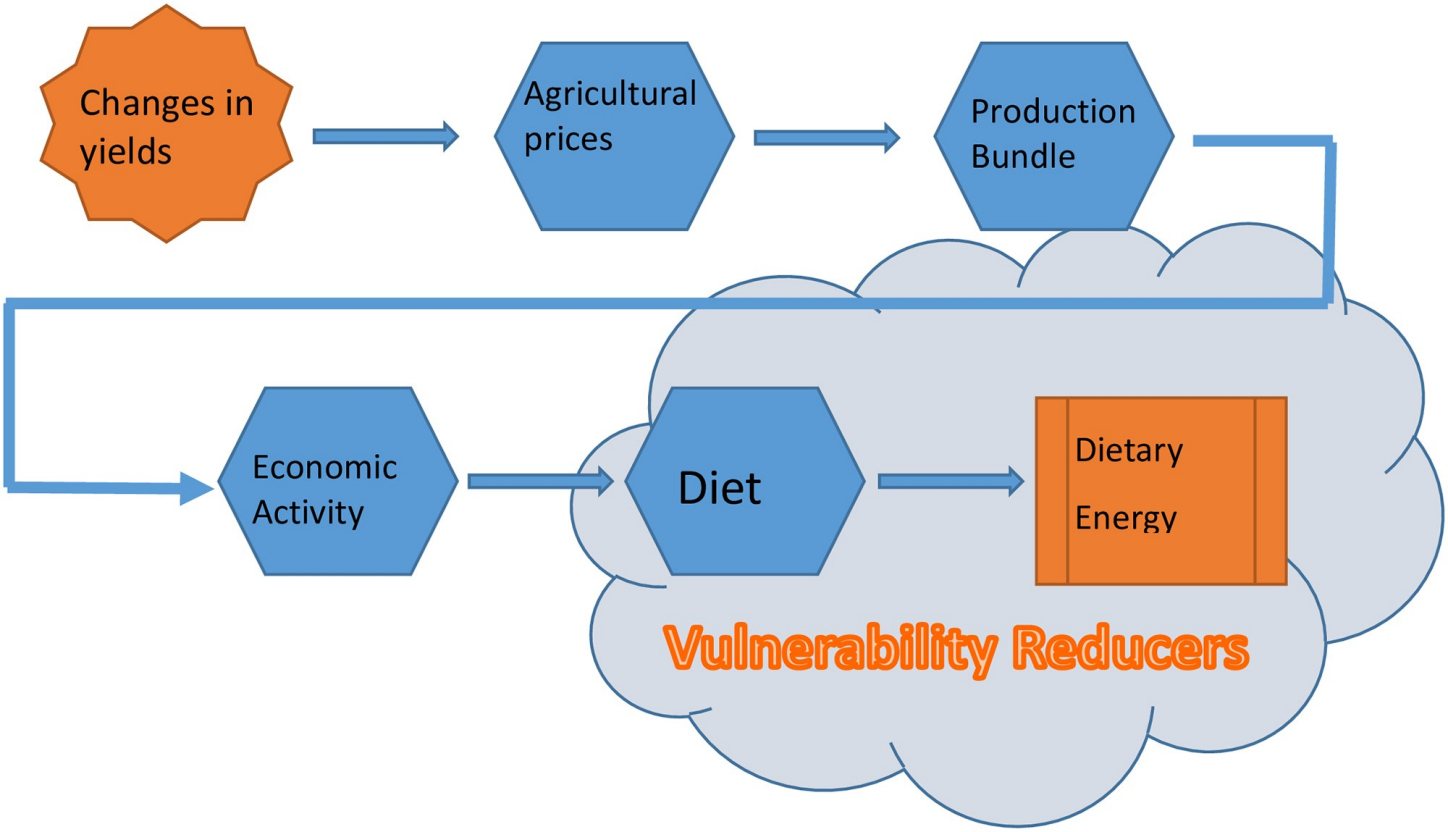
Mediators between vulnerability and climate change.

Moreover, in Peru and elsewhere in the world, the impact of changes in agricultural yields on welfare are mediated by the markets and mitigating activities the households perform. CC, as modeled in this study, impacts household incomes through the value of agricultural production that can increase or diminish as a result of climate driven changes in agricultural productivity. This means that the impact that climate change could have on salaries (agricultural) are ignored, but at the rural level only 8% of income is derived from agricultural salaries, which shows that at the aggregated level (not necessarily locally), the impact of changes in agricultural salaries on vulnerability would be greatly reduced. However, as shown in Fig 3, there are many mediators between these impacts on agricultural productivity and the final welfare outcome.

First are agricultural markets. When the agricultural supply expands or contracts due to changes in productivity, the changes in demand are mediated by prices. For example, if supply falls 20%, prices should increase, moderating the negative impact of the fall in production on incomes. Later, if a crop shows a permanent reduction in its yield, the rational response of the farmer is to change the crop. It is for that reason that the selection of an optimum bundle of crops is another mediating mechanism on the impact of changes in productivity of crops. If agriculture in general has lost competitiveness due to negative impacts on its productivity, the natural response of a household is to change employment decisions and to increase the effort spent on non-agricultural activities, to the detriment of time spent on agricultural activities. Finally, if we look specifically at impacts on undernourishment, households have another mechanism to mitigate negative impacts, which is to alter their diets by consuming cheaper calories (generally cereals) and less expensive calories (fruits, vegetables, and animal proteins).

On the other hand, all of these mechanisms that mitigate the impact of climate on wellbeing, those of the market and of individual choice, transpire in a socioeconomic context in which there are factors that reduce and increase vulnerability. We know, for example, that the ownership of economic assets helps reduce vulnerability as they act as financial buffers that cushion negative shocks, be them climatic or of a different nature.

This paper has established that CC has a moderate impact on vulnerability to food insecurity. Given that we have all the information, we close this analysis by opening the discussion of what are therefore, the main drivers of vulnerability in this context of CC. We open this question with an estimation of the determinants of vulnerability with a probit model of the binary outcome, vulnerable or not, against the same set of household-head characteristics, household characteristics, and location features used to estimate the caloric consumption model above (see Table 6). The full model is presented in S5 Table, while Table 9, summarizes the variables with the highest marginal impact on vulnerability. It is important know which factors are most important in determining vulnerability to hunger, as some of these characteristics can be impacted with public policy, to support vulnerable families in dealing with CC as well as other shocks that rural families face each season.

**Table 9:** Marginal effects of selected indicators on vulnerability.

Characteristic	Marginal Prob. Impact
Economic Dependence	24.9
Agricultural household binary	-24.4
Mountains	18.1
Number of people in the household	12.3
Participate in soup kitchens	-11.5
Infrastructure index	-7.9
Rainforest	7.5
School dropout	7.2

Note: All effects significant at p-value < .001.

Source: Summary of main results of a full probit model available in S5 Table.

The table of the main determinants of vulnerability offers some clear policy access-points. The two primary characteristics, rate of economic dependence of the household and agricultural household (the rate of economic dependence is the ratio of household members that do not work over to the total number of people in the household), are the result of rural markets that are not completely developed. If the labor markets and agricultural products markets functioned perfectly (instead of working imperfectly due to communication, information, and/or infrastructure failures), it should not be significant whether the household was or was not agricultural, but only the quantity of perceived income (monetary and non-monetary) would matter. In this case as we are controlling for income sources (farm and non-farm), in the probit model, the large impact of being an agricultural household is larger than the value of all the agricultural income generated. If labor markets worked perfectly farmers would assign effort to farm and non-farm activities such as to equate marginal returns, and it would not matter the source of income. The economic dependence (proportion of the members of the household who don’t earn income) is an indicator of a lack of employment, which could be due to little job creation or poor integration of labor markets, for example due to poor infrastructure and communications, and the stage of the demographic transition of Peru. Household size is more difficult to interpret, it can be an indication that rural households prefer to have larger households, or that they do not have access to family planning services. There is an extensive discussion of this in the literature, see for example, (López and Valdés 2000). Infrastructure availability (index), living in the rainforest, and sierra regions, reinforces the message about the importance of infrastructure and where it is most needed: in the Andes and the Amazonian rainforest. The public policies of social protection are also important, when they are well targeted (e.g. soup kitchens), and education is important but requires a focalized intervention on the most vulnerable which many times respond with school absenteeism as a household strategy (to supplement labor shortages in the household).

This final exploratory analysis is perhaps the most important message of this study: as it aims to study the impact of climate change on wellbeing, it concludes that we must continue emphasizing the policy lessons that have been highlighted in the literature before global interest in CC emerged. With or without climate change, to fight hunger in Peru the focus must continue on increasing investment on rural public goods (infrastructure and education) with a focalized effort on the most lagging places (especially in the sierra region), and continue with the improved targeting of social protection programs.

## 6. Conclusions

Perhaps one of the main findings of this study is that impacts on yields, even large ones, produced by changing climate patterns do not necessarily translate to large impacts in population suffering food insecurity. This result has a direct implication for food security policies in the context of climate change. Advances in crop sciences, like improvements in heat and drought tolerance of major crops represent a very important contribution to the resilience of global agri-food systems. These scientific advances should also help in the expansion of the agricultural frontier as both yields and area continue to contribute to the required increases of food production towards 2050 and beyond [37]. However, our findings suggest that these policies are not very efficient in improving food security at the household-level. This research shows that the best way to reduce poor households’ vulnerability to climate change is to affect those variables that reduce vulnerability more broadly. In the case of Peru, these policies are those that have been generally emphasized in the literature before the rise in CC awareness, like investment in public goods (education and infrastructure) as a way to alleviate market imperfections, while supporting the most vulnerable population with well-targeted social protection schemes. In other word, CC does not appear to change the best strategies to combat vulnerability to hunger, and these strategies are country/context specific.

In thinking about policies that combat food insecurity in the context of CC it is important to note the limitations of the modeling efforts, like the one presented here that link climate information with agricultural production from household surveys. On the one hand there are climate modeling limitations, which are highly relevant for modeling agricultural response. Climate change models, like ESM used in this report, do not consider changes in extreme events patterns (like hurricanes, El Niño events, or other). These extreme events may revert estimated impacts on yields, and likely require different policy responses than those considered here. The way that climate impacts household-level agricultural productivity can also be made more precise. For example, in the case of Peru, crops can be divided by eco-region-specific crops, and separate yield equations could be estimated. Finally, it is important to consider the effects of CC downstream from agriculture. The model presented here does not consider sector-wide employment impacts of CC, effects on employment and wages in the non-agricultural sectors. The latter could be significant, specifically through agricultural growth multiplier effects in less developed countries where agriculture is a large sector of GDP.

## Supporting information

S1 File Data and program in STATA to replicate results.zip. All tables in this paper can be replicated using STATA v12 and later with the dataset and program included here.
(ZIP)

S1 Table Different specifications of the climate vector. (DOCX)

S2 Table Climate variables correlation. (DOCX)

S3 Table Estimation results of agricultural productivity (yields). (DOCX)

S4 Table Estimated results of determinants of caloric consumption and the variance of caloric consumption. (DOCX)

S5 Table Probability of being a vulnerable household (probit model- year 2012). (DOCX)

S6 Table Predicted climate: Average (2048–50) by geographic domain: CanES ESM. (DOCX)

S7 Table Predicted climate: Average (2048–50) by geographic domain: CNR ESM. (DOCX)

S8 Table Predicted climate: Average (2048–50) by geographic domain: MPI ESM. (DOCX)

S9 Table Effect of CNR climate simulations on yields. (DOCX)

S10 Table Effect of MPI climate simulations on yields. (DOCX)

S11 Table Effect of climate simulations on mean caloric consumption: CanES model. (DOCX)

S12 Table Effect of climate simulations on mean caloric consumption: CNR model. (DOCX)

S13 Table Effect of climate simulations on mean caloric consumption: MPI model. (DOCX)

S14 Table Effect of climate simulations on vulnerability: CNR model. (DOCX)

S15 Table Effect of climate simulations on vulnerability: MPI model. (DOCX)
