# Supplementary material for: De-climatizing food security: Lessons from climate change micro-simulations in Peru
Source: PLoS One. 2019 Sep 27;14(9):e0222483. doi: 10.1371/journal.pone.0222483 (PMC6764669; doi:10.1371/journal.pone.0222483)
Supplement: S1 Table — (DOCX) [file pone.0222483.s002.docx]

**Table S1. Different Specifications of the Climate Vector**

|  | **Model** | **Log Likelihood** | **R2** | **Adjusted R2** | **AIC** | **BIC** | **Climate R2 (%)** |
| --- | --- | --- | --- | --- | --- | --- | --- |
| 1 | No Climate Vars. | -41258.1 | 0.707 | 0.7063 | 82624.1 | 83077.3 | 0 |
| 2 | Annual Means (Tmax, Tmean, Precip.) | -41063.6 | 0.708 | 0.7071 | 82241.2 | 82719.3 | 5.6 |
| 3 | Annual Means and Coeff. of Variability | -41040.3 | 0.708 | 0.7075 | 82200.5 | 82703.8 | 5.9 |
| 4 | Annual Means and Coeff. of Variability and Ecoregion | -40818.5 | 0.712 | 0.7114 | 81781.0 | 82385.0 | 9.4 |
| 5 | Rolling Mean and Annual Deviation (Tmax, Tmean, Precip., Precip. Seasonality) and Ecoregion | -40562.4 | 0.717 | 0.7159 | 81280.7 | 81935.0 | 10.2 |
| 6 | Model 5 with Tmax-Altitude Interaction | -40542.1 | 0.717 | 0.7162 | 81252.1 | 81956.7 | 10.4 |
| 7 | Model 5 with Tmax-Altitude (Sierra Only) | -40554.1 | 0.717 | 0.7160 | 81268.1 | 81939.1 | 10.3 |
| 8 | Model 5 with Tmean-Altitude Interaction | -40532.5 | 0.717 | 0.7163 | 81233.1 | 81937.7 | 10.4 |
| 9 | Model 5 with Tmean-Alt. (Sierra Only) | -40544.8 | 0.717 | 0.7162 | 81249.5 | 81920.6 | 10.3 |
| 10 | Model 5 with (Tmax & Tmean)-Alt. Interaction | -40520.0 | 0.717 | 0.7165 | 81220.0 | 81974.9 | 10.7 |
| 11 | Model 5 wit (Tmax & Tmean)-Alt. (Sierra only) | -40542.4 | 0.717 | 0.7162 | 81248.7 | 81936.5 | 10.4 |

Notes: The last column indicates the portion of total R-squared contributed by the climate vector, according to the Shapley decomposition, see text
