# Supplementary material for: De-climatizing food security: Lessons from climate change micro-simulations in Peru
Source: PLoS One. 2019 Sep 27;14(9):e0222483. doi: 10.1371/journal.pone.0222483 (PMC6764669; doi:10.1371/journal.pone.0222483)
Supplement: S2 Table — (DOCX) [file pone.0222483.s003.docx]

**Table S2. Climate Variables Correlation**

|  | Maximum Temperature | | | | | | Max Temperature Year Deviation | | | | | | Mean Temperature | | | | | | Mean Temperature Year Dev. | | | | | |
| --- | --- | --- | --- | --- | --- | --- | --- | --- | --- | --- | --- | --- | --- | --- | --- | --- | --- | --- | --- | --- | --- | --- | --- | --- |
|  | Coast | | Mountains | | Rainforest | | Coast | | Mountains | | Rainforest | | Coast | | Mountains | | Rainforest | | Coast | | Mountains | | Rainforest | |
|  |  |  |  |  |  |  |  |  |  |  |  |  |  |  |  |  |  |  |  |  |  |  |  |  |
| Maximum temp. (cumul. MA) - Coast |  |  |  |  |  |  |  |  |  |  |  |  |  |  |  |  |  |  |  |  |  |  |  |  |
| Mountains | -0.543 | * |  |  |  |  |  |  |  |  |  |  |  |  |  |  |  |  |  |  |  |  |  |  |
| Rainforest | -0.212 | * | -0.667 | * |  |  |  |  |  |  |  |  |  |  |  |  |  |  |  |  |  |  |  |  |
| Maximum temp. deviation – Coast | 0.810 | * | -0.439 | * | -0.171 | * |  |  |  |  |  |  |  |  |  |  |  |  |  |  |  |  |  |  |
| Mountains | -0.333 | * | 0.551 | * | -0.409 | * | -0.269 | * |  |  |  |  |  |  |  |  |  |  |  |  |  |  |  |  |
| Rainforest | -0.175 | * | -0.552 | * | 0.812 | * | -0.142 | * | -0.339 | * |  |  |  |  |  |  |  |  |  |  |  |  |  |  |
| Average temp. (cumul. MA) – Coast | 0.999 | * | -0.541 | * | -0.211 | * | 0.807 | * | -0.332 | * | -0.175 | * |  |  |  |  |  |  |  |  |  |  |  |  |
| Mountains | -0.491 | * | 0.962 | * | -0.603 | * | -0.396 | * | 0.463 | * | -0.499 | * | -0.488 | * |  |  |  |  |  |  |  |  |  |  |
| Rainforest | -0.209 | * | -0.659 | * | 0.997 | * | -0.169 | * | -0.404 | * | 0.799 | * | -0.208 | * | -0.595 | * |  |  |  |  |  |  |  |  |
| Average temp. deviation – Coast | 0.812 | * | -0.436 | * | -0.170 | * | 0.925 | * | -0.268 | * | -0.141 | * | 0.815 | * | -0.394 | * | -0.168 | * |  |  |  |  |  |  |
| Mountains | -0.350 | * | 0.606 | * | -0.431 | * | -0.283 | * | 0.907 | * | -0.357 | * | -0.349 | * | 0.541 | * | -0.425 | * | -0.282 | * |  |  |  |  |
| Rainforest | -0.169 | * | -0.531 | * | 0.773 | * | -0.136 | * | -0.326 | * | 0.943 | * | -0.168 | * | -0.480 | * | 0.757 | * | -0.136 | * | -0.343 | * |  |  |
| Precipitation (cumul. MA) - Coast | 0.943 | * | -0.504 | * | -0.196 | * | 0.789 | * | -0.309 | * | -0.163 | * | 0.951 | * | -0.455 | * | -0.194 | * | 0.809 | * | -0.325 | * | -0.157 | * |
| Mountains | -0.457 | * | 0.804 | * | -0.562 | * | -0.370 | * | 0.507 | * | -0.466 | * | -0.455 | * | 0.731 | * | -0.555 | * | -0.368 | * | 0.596 | * | -0.448 | * |
| Rainforest | -0.199 | * | -0.627 | * | 0.958 | * | -0.161 | * | -0.385 | * | 0.773 | * | -0.198 | * | -0.566 | * | 0.964 | * | -0.160 | * | -0.405 | * | 0.712 | * |
| Precipitation deviation - Coast | -0.292 | * | 0.155 | * | 0.060 |  | -0.288 | * | 0.095 | * | 0.050 |  | -0.299 | * | 0.140 | * | 0.060 |  | -0.199 | * | 0.100 | * | 0.048 |  |
| Mountains | -0.107 |  | 0.200 | * | -0.131 | * | -0.086 |  | 0.094 | * | -0.108 | * | -0.106 | * | 0.205 | * | -0.129 | * | -0.086 |  | 0.207 | * | -0.104 | * |
| Rainforest | -0.091 |  | -0.286 | * | 0.436 | * | -0.073 |  | -0.176 | * | 0.288 | * | -0.090 |  | -0.259 | * | 0.432 | * | -0.073 |  | -0.185 | * | 0.288 | * |
| Seasonality of precip. (cumul. MA) – Coast | 0.982 | * | -0.534 | * | -0.208 | * | 0.814 | * | -0.327 | * | -0.172 | * | 0.982 | * | -0.482 | * | -0.206 | * | 0.824 | * | -0.344 | * | -0.166 | * |
| Mountains | -0.531 | * | 0.904 | * | -0.653 | * | -0.429 | * | 0.586 | * | -0.541 | * | -0.529 | * | 0.783 | * | -0.645 | * | -0.427 | * | 0.571 | * | -0.520 | * |
| Rainforest | -0.208 | * | -0.654 | * | 0.939 | * | -0.168 | * | -0.402 | * | 0.805 | * | -0.207 | * | -0.591 | * | 0.911 | * | -0.167 | * | -0.423 | * | 0.795 | * |
| Seasonality of precip. Deviation - Coast | -0.146 | * | 0.099 | * | 0.039 |  | -0.134 | * | 0.061 |  | 0.032 |  | -0.113 | * | 0.089 |  | 0.038 |  | -0.046 |  | 0.064 |  | 0.031 |  |
| Mountains | -0.028 |  | 0.027 |  | -0.034 |  | -0.022 |  | 0.031 |  | -0.028 |  | -0.028 |  | 0.003 |  | -0.034 |  | -0.022 |  | 0.086 |  | -0.027 |  |
| Rainforest | -0.038 |  | -0.120 | * | 0.162 | * | -0.031 |  | -0.074 |  | 0.077 |  | -0.038 |  | -0.109 | * | 0.157 | * | -0.031 |  | -0.078 |  | 0.083 |  |

**Table S3. Climate Variables Correlation (cont.)**

|  | Cumulative Average Precipitation | | | | | | Precipitation Year Deviation | | | | | | Rainfall Seasonality | | | | | | Rainfall Seasonality Year Dev. | | | | | |
| --- | --- | --- | --- | --- | --- | --- | --- | --- | --- | --- | --- | --- | --- | --- | --- | --- | --- | --- | --- | --- | --- | --- | --- | --- |
|  | Coast | | Mountains | | Rainforest | | Coast | | Mountains | | Rainforest | | Coast | | Mountains | | Rainforest | | Coast | | Mountains | | Rainforest | |
|  |  |  |  |  |  |  |  |  |  |  |  |  |  |  |  |  |  |  |  |  |  |  |  |  |
| Precipitation (cumul. MA) - Coast |  |  |  |  |  |  |  |  |  |  |  |  |  |  |  |  |  |  |  |  |  |  |  |  |
| Mountains | -0.424 | * |  |  |  |  |  |  |  |  |  |  |  |  |  |  |  |  |  |  |  |  |  |  |
| Rainforest | -0.185 | * | -0.528 | * |  |  |  |  |  |  |  |  |  |  |  |  |  |  |  |  |  |  |  |  |
| Precipitation deviation - Coast | -0.268 | * | 0.130 | * | 0.057 |  |  |  |  |  |  |  |  |  |  |  |  |  |  |  |  |  |  |  |
| Mountains | -0.099 | * | 0.406 | * | -0.123 | * | 0.030 |  |  |  |  |  |  |  |  |  |  |  |  |  |  |  |  |  |
| Rainforest | -0.084 |  | -0.241 | * | 0.536 | * | 0.026 |  | -0.056 |  |  |  |  |  |  |  |  |  |  |  |  |  |  |  |
| Seasonality of precip. (cumul. MA) – Coast | 0.905 | * | -0.450 | * | -0.196 | * | -0.314 | * | -0.105 | * | -0.089 |  |  |  |  |  |  |  |  |  |  |  |  |  |
| Mountains | -0.493 | * | 0.641 | * | -0.614 | * | 0.151 | * | 0.096 | * | -0.280 | * | -0.522 | * |  |  |  |  |  |  |  |  |  |  |
| Rainforest | -0.193 | * | -0.551 | * | 0.849 | * | 0.059 |  | -0.128 | * | 0.404 | * | -0.204 | * | -0.641 | * |  |  |  |  |  |  |  |  |
| Seasonality of precip. Deviation - Coast | -0.013 |  | 0.083 |  | 0.036 |  | 0.117 | * | 0.019 |  | 0.017 |  | -0.109 | * | 0.097 | * | 0.038 |  |  |  |  |  |  |  |
| Mountains | -0.026 |  | 0.199 | * | -0.032 |  | 0.008 |  | 0.295 | * | -0.015 |  | -0.027 |  | -0.015 |  | -0.033 |  | 0.005 |  |  |  |  |  |
| Rainforest | -0.036 |  | -0.102 | * | 0.075 |  | 0.011 |  | -0.024 |  | 0.151 | * | -0.038 |  | -0.118 | * | 0.208 | * | 0.007 |  | -0.006 |  |  |  |

Notes: A star (*) indicates correlation is different from zero with *p*-val.<0.01 . 765 observations, 196 provinces times 4 survey years, minus one rainforest province not sampled by household survey on 2005.
