# Supplementary material for: De-climatizing food security: Lessons from climate change micro-simulations in Peru
Source: PLoS One. 2019 Sep 27;14(9):e0222483. doi: 10.1371/journal.pone.0222483 (PMC6764669; doi:10.1371/journal.pone.0222483)
Supplement: S3 Table — (DOCX) [file pone.0222483.s004.docx]

**Table S3. Estimation Results of Agricultural Productivity (Yields)**

| Dependent Variable ln (index of agricultural production/hectares of operated land) | | | | Coefficient | *t*-stat |
| --- | --- | --- | --- | --- | --- |
| I: Agricultural Inputs | | ln hectares of operated land | | -0.677^***^ | 35.99 |
|  |  | ln number of agricultural workers | | -0.0016 | 0.09 |
|  |  | ln spending on agricultural labor | | 0.0385^***^ | 14.92 |
|  |  | ln spending on agricultural variable inputs | | 0.202^***^ | 31.17 |
|  |  | ln spending on livestock variable inputs | | 0.0158^***^ | 4.35 |
|  |  | Irrigation: drip | | -0.0388 | 1.33 |
|  |  | Irrigation: gravity-fed | | -0.0477^***^ | 2.60 |
|  |  | Irrigation: wells | | 0.113^**^ | 2.04 |
| K: Physical Capital | | Horses | | -0.0076 | 0.55 |
|  |  | Cows | | -0.0939^***^ | 6.28 |
|  |  | Llamas | | -0.0733^*^ | 1.85 |
|  |  | ln infrastructure index | | 0.101^***^ | 9.08 |
|  |  | Motorcycle dummy | | 0.0619^**^ | 2.42 |
|  |  | Car or truck dummy | | 0.155^***^ | 5.29 |
| FC: Farmer Characteristics | | Male head of household dummy | | 0.0996^***^ | 6.08 |
|  |  | ln years of schooling of head of household | | 0.0257^***^ | 4.03 |
|  |  | ln age of head of household | | 1.313^***^ | 3.69 |
|  |  | ln age of head of household squared | | -0.179^***^ | 3.86 |
|  |  | Head of household speaks indigenous language dummy | | -0.0796^***^ | 3.40 |
| HC: Household Characteristics | | ln number of people in the household | | 0.224^***^ | 10.64 |
|  |  | Percentage of people in the household who do not work | | -0.201^***^ | 5.15 |
|  |  | Share of agricultural income in total income | | 0.0119^***^ | 36.14 |
| Cl: Climatic Variables | | Maximum temperature (moving average) - Coast | | -0.107 | 1.54 |
|  |  | Maximum temperature (moving average) - Mountains | | -0.124^***^ | 3.14 |
|  |  | Maximum temperature (moving average) - Rainforest | | -0.0666 | 0.74 |
|  |  | Maximum temperature deviation period - Coast | | 0.354^***^ | 2.89 |
|  |  | Maximum temperature deviation period - Mountains | | -0.0477 | 0.76 |
|  |  | Maximum temperature deviation period - Rainforest | | -0.573^***^ | 5.39 |
|  |  | Average temperature (moving average) - Coast | | 0.162^***^ | 2.70 |
|  |  | Average temperature (moving average) - Mountains | | 0.139^***^ | 3.86 |
|  |  | Average temperature (moving average) - Rainforest | | 0.084 | 1.08 |
|  |  | Average temperature deviation period - Coast | | -0.320^**^ | 2.56 |
|  |  | Average temperature deviation period - Mountains | | 0.2097 | 1.82 |
|  |  | Average temperature deviation period - Rainforest | | 0.820^***^ | 9.04 |
|  |  | Precipitation (moving average) - Coast | | -0.0002 | 0.7 |
|  |  | Precipitation (moving average) - Mountains | | 0.00027^***^ | 2.74 |
|  |  | Precipitation (moving average) - Rainforest | | 0.000158^***^ | 2.74 |
|  |  | Precipitation deviation period - Coast | | 0.000336^*^ | 1.71 |
|  |  | Precipitation deviation period - Mountains | | -0.00076^***^ | 7.55 |
|  |  | Precipitation deviation period - Rainforest | | -0.00021^**^ | 2.27 |
|  |  | Index of seasonal precipitation (moving average) - Coast | | -0.1489 | 0.48 |
|  |  | Index of seasonal precipitation (moving average) - Mountains | | 0.1228 | 0.44 |
|  |  | Index of seasonal precipitation (moving average) - Rainforest | | 2.7789^***^ | 3.72 |
|  |  | Deviation period of index of seasonal precipitation - Coast | | 0.0911 | 0.46 |
|  |  | Deviation period of index of seasonal precipitation - Mountains | | 0.5681^***^ | 3.42 |
|  |  | Deviation period of index of seasonal precipitation - Rainforest | | 1.7756^***^ | 5.73 |
|  |  | Average temperature (moving ave.) – Mountains x Altitude | | -1.1E-06 | 0.55 |
|  |  | Average temperature deviation period – Mountains x Altitude | | 9.4E-05^***^ | 3.04 |
| G , FE: Geographic characteristics and fixed effects | | Altitude | | -5.75e-05^***^ | 2.72 |
|  |  | Latitude* | | 0.0015 | 0.36 |
|  |  | Year dummies**: 2007 | | 0.276^***^ | 9.19 |
|  |  | 2010 | | 0.421^***^ | 9.40 |
|  |  | 2012 | | 0.385^***^ | 10.57 |
|  |  | Eco-region dummies***: Mountains | | 0.168 | 0.17 |
|  |  | Rainforest | | -1.675 | 1.14 |
|  |  | Department dummies: Ancash | | -0.069 | 1.00 |
|  |  | Apurimac | | -0.0278 | 0.33 |
|  |  | Arequipa | | 0.541^***^ | 4.69 |
|  |  | Ayacucho | | -0.0094 | 0.12 |
|  |  | Cajamarca | | -0.629^***^ | 11.44 |
|  |  | Callao | | 0.687^*^ | 1.83 |
|  |  | Cusco | | 0.1155 | 1.27 |
|  |  | Huancavelica | | -0.1908^**^ | 2.47 |
|  |  | Huanuco | | -0.2583^***^ | 4.02 |
|  |  | Ica | | 0.4158^***^ | 2.98 |
|  |  | Junin | | 0.2343^***^ | 3.15 |
|  |  | La Libertad | | -0.131^*^ | 1.65 |
|  |  | Lambayeque | | -0.504^***^ | 4.92 |
|  |  | Lima | | 0.4724^***^ | 3.14 |
|  |  | Loreto | | -0.053 | 0.58 |
|  |  | Madre De Dios | | -0.4581^***^ | 3.23 |
|  |  | Moquegua | | 0.570^***^ | 4.67 |
|  |  | Pasco | | 0.1402^*^ | 1.79 |
|  |  | Piura | | -0.267^***^ | 2.88 |
|  |  | Puno | | 0.317^***^ | 3.44 |
|  |  | San Martin | | -0.134^**^ | 2.12 |
|  |  | Tacna | | 0.635^***^ | 5.04 |
|  |  | Tumbes | | -0.314^**^ | 2.19 |
|  |  | Ucayali | | 0.1066 | 1.25 |
|  | | Observations | | 32,466 |  |
|  | | R-squared | | 0.717 |  |
|  | | Initial Log-likelihood | | -61027 |  |
|  | | Final Log-likelihood | | -40544 |  |
|  | | AIC | | 81249 |  |
|  | | BIC | | 81920 |  |
| *t*-stat estimated with robust standard errors by survey PSU. | |  |  |  |  |
|  | |  |  |  |  |

Notes: i) The effect of longitude is implicitly captured by the three eco-region dummies; ii) omitted year 2005; iii) omitted eco-region coast; iv) omitted department Amazonas
