# Supplementary material for: De-climatizing food security: Lessons from climate change micro-simulations in Peru
Source: PLoS One. 2019 Sep 27;14(9):e0222483. doi: 10.1371/journal.pone.0222483 (PMC6764669; doi:10.1371/journal.pone.0222483)
Supplement: S4 Table — (DOCX) [file pone.0222483.s005.docx]

**Table S4. Estimated results of determinants of caloric consumption and the variance of caloric consumption.**

| **Name of variables** | | **Caloric Consumption** | | **Consumption Variance** |
| --- | --- | --- | --- | --- |
| Climatic risk | | -0.00434*** | | -0.0117*** |
|  | | (4.72) | | (4.05) |
| Agricultural household dummy | | 0.115*** | | -0.468*** |
|  | | (9.66) | | (13.44) |
| Male head of household dummy | | 0.00777 | | -0.122*** |
|  | | (0.85) | | (4.20) |
| ln schooling years of the head of household | | 0.00208*** | | -0.00541** |
|  | | (3.29) | | (2.45) |
| ln age of the head of household | | 0.0123*** | | -0.0310*** |
|  | | (11.20) | | (10.12) |
| ln age of the head of household squared | | -0.000134*** | | 0.000326*** |
|  | | (12.80) | | (11.29) |
| Head of household married or cohabitating dummy | | -0.0115** | | -0.0949*** |
|  | | (2.08) | | (4.98) |
| Head of household widowed dummy | | 0.00506 | | 0.108*** |
|  | | (0.49) | | (3.83) |
| Head of household speaks an indigenous language dummy | | -0.00761 | | -0.00543 |
|  | | (0.97) | | (0.22) |
| Percent of people in the household who do not work | | -0.231*** | | 0.165*** |
|  | | (20.80) | | (5.32) |
| ln household size | | -0.0813*** | | -0.0813*** |
|  | | (43.42) | | (14.27) |
| ln no. women in the household | | -0.0977*** | | -0.314*** |
|  | | (8.17) | | (8.75) |
| Average schooling years of the household | | -0.00226** | | 0.00858*** |
|  | | (2.25) | | (2.69) |
| Infrastructure index | | 0.0473*** | | -0.172*** |
|  | | (10.22) | | (11.27) |
| Assets index | | 0.0167*** | | -0.107*** |
|  | | (2.97) | | (5.43) |
| School dropout member dummy | | -0.0415*** | | 0.0737*** |
|  | | (6.23) | | (3.15) |
| Participates in Vaso de Leche program dummy | | -0.0256*** | | -0.132*** |
|  | | (4.96) | | (7.48) |
| Participates in soup kitchens dummy | | 0.0784*** | | -0.158*** |
|  | | (8.47) | | (4.79) |
| Share of agricultural income in total income | | -0.000155** | | 0.000178 |
|  | | (2.40) | | (0.84) |
| Index of value of agricultural production (agricultural | | 0.0261*** | | 0.0298*** |
| Income - predicted) | | (14.67) | | (8.33) |
| Non-agricultural income | | 2.68e-05*** | | 1.99e-05*** |
|  | | (21.47) | | (8.54) |
| Year dummies: 2007 | | -0.104*** | | 0.352*** |
|  | | (8.39) | | (9.25) |
| 2010 | | -0.0487*** | | 0.0714 |
|  | | (3.26) | | (1.50) |
| 2012 | | 0.0157** | | -0.113*** |
|  | | (2.34) | | (5.06) |
| Eco-region dummies: Sierra – Andes Region | | -0.0784*** | | 0.00254 |
|  | | (10.55) | | (0.11) |
| Selva – Rainforest Region | | -0.0288*** | | 0.0479* |
|  | | (3.73) | | (1.95) |
| Department dummies: Ancash | | -0.0131 | | 0.0742 |
|  | | (0.82) | | (1.44) |
| Apurimac | | -0.0610*** | | -0.0779 |
|  | | (3.76) | | (1.48) |
| Ayacucho | | -0.0526** | | 0.196*** |
|  | | (2.40) | | (3.12) |
| Cajamarca | | -0.0517*** | | -0.0225 |
|  | | (3.28) | | (0.44) |
| Callao | | -0.0688*** | | -0.0995** |
|  | | (5.57) | | (2.37) |
| Cusco | | 0.0299* | | -0.0399 |
|  | | (1.92) | | (0.78) |
| Huancavelica | | -0.123*** | | 0.0585 |
|  | | (7.66) | | (1.14) |
| Huanuco | | -0.0739*** | | 0.204*** |
|  | | (5.04) | | (4.38) |
| Ica | | -0.0538** | | 0.121* |
|  | | (2.38) | | (1.73) |
| Junín | | -0.0268** | | -0.281*** |
|  | | (1.97) | | (5.84) |
| La Libertad | | -0.0218 | | 0.0853 |
|  | | (1.20) | | (1.49) |
| Lambayeque | | 0.0271 | | -0.165** |
|  | | (1.41) | | (2.44) |
| Lima | | -0.124*** | | 0.230*** |
|  | | (5.47) | | (3.54) |
| Loreto | | -0.113*** | | 0.168*** |
|  | | (7.83) | | (3.42) |
| Madre De Dios | | 0.116*** | | -0.438*** |
|  | | (7.60) | | (7.82) |
| Moquegua | | -0.162*** | | 0.265*** |
|  | | (7.10) | | (4.18) |
| Pasco | | -0.247*** | | 0.152*** |
|  | | (14.77) | | (2.93) |
| Piura | | -0.0538*** | | -0.0151 |
|  | | (2.99) | | (0.25) |
| Puno | | -0.0138 | | -0.332*** |
|  | | (0.89) | | (6.37) |
| San Martin | | 0.0261* | | 0.149*** |
|  | | (1.91) | | (3.16) |
| Tacna | | -0.149*** | | 0.290*** |
|  | | (6.34) | | (4.36) |
| Tumbes | | -0.0446* | | 0.0692 |
|  | | (1.93) | | (0.90) |
| Ucayali | | 0.0887*** | | -0.0139 |
|  | | (5.64) | | (0.26) |
| Constant | | 7.860*** | | -0.0997 |
|  | | (229.20) | | (1.01) |
| Observations | | 35,358 | | 35,358 |
| Robust z-statistics in parentheses | |  |  |  |
| *** p<0.01, ** p<0.05, * p<0.1 | |  |  |  |

Notes: i) omitted year 2005; ii) omitted eco-region coast; ii) omitted department Amazonas
