# Supplementary material for: De-climatizing food security: Lessons from climate change micro-simulations in Peru
Source: PLoS One. 2019 Sep 27;14(9):e0222483. doi: 10.1371/journal.pone.0222483 (PMC6764669; doi:10.1371/journal.pone.0222483)
Supplement: S5 Table — (DOCX) [file pone.0222483.s006.docx]

**Table S5. Probability of being a vulnerable household (Probit Model- year 2012).**

| **Variable** | | **Coefficient** | **Marginal Effect** | |
| --- | --- | --- | --- | --- |
| Climatic Risk | | 0.0817^***^ | 0.00736^***^ | |
|  | | (7.35) | (7.56) | |
| Dummy agricultural household | | -2.707^***^ | -0.2437^***^ | |
|  | | (17.75) | (19.80) | |
| Dummy male head of household | | -0.0315 | -0.00283 | |
|  | | (0.35) | (0.35) | |
| Schooling years of the head of household | | -0.0660^***^ | -0.00594 | |
|  | | (9.19) | (9.35) | |
| Age of the head of household | | -0.0966^***^ | -0.0087^***^ | |
|  | | (7.39) | (7.37) | |
| Age of the head of household squared | | 0.00112^***^ | 0.0001^***^ | |
|  | | (9.42) | (9.41) | |
| Dummy head of household married or cohabitating | | -0.0477 | -0.0043 | |
|  | | (0.74) | (0.74) | |
| Dummy head of household widowed | | 0.155 | 0.0140 | |
|  | | (1.33) | (1.33) | |
| Dummy head of household speaks an indigenous tongue language | | 0.186^**^ | 0.01675^**^ | |
|  | | (2.15) | (2.16) | |
| Percent of people in the household who do not work (Economic | | 2.760^***^ | 0.2485^***^ | |
| dependency) | | (19.76) | (22.00) | |
| Household size | | 1.361^***^ | 0.1226^***^ | |
|  | | (35.47) | (65.46) | |
| No. women in the household | | -0.166 | -0.01494 | |
|  | | (1.18) | (1.18) | |
| Average schooling years of the household | | 0.154^***^ | 0.01385^***^ | |
|  | | (12.22) | (12.64) | |
| Infrastructure index | | -0.872^***^ | -0.0785^***^ | |
|  | | (17.05) | (19.07) | |
| Assets index | | -0.516^***^ | -0.0465^***^ | |
|  | | (7.72) | (7.76) | |
| School dropout member dummy | | 0.804^***^ | 0.0724^***^ | |
|  | | (10.89) | (11.46) | |
| Participates in Vaso de Leche program | | -0.642^***^ | -0.0578^***^ | |
|  | | (10.54) | (10.98) | |
| Participates in soup kitchens | | -1.277^***^ | -0.11497^***^ | |
|  | | (11.14) | (11.38) | |
| Share of agricultural income in total income | | 0.00218^*^ | 0.0002^*^ | |
|  | | (1.71) | (1.71) | |
| Index of value of agricultural production (agricultural | | -0.231^***^ | -0.02078^***^ | |
| Income - predicted) | | (5.23) | (5.28) | |
| Non-agricultural income | | -0.000387^***^ | -0.000035^***^ | |
|  | | (18.49) | (20.17) | |
| Sierra – Andes Region | | 2.012^***^ | 0.1812^***^ | |
|  | | (14.68) | (15.44) | |
| Selva – Rainforest Region | | 0.837^***^ | 0.0754^***^ | |
|  | | (4.98) | (5.01) | |
| Ancash | | 0.225 | 0.0203 | |
|  | | (1.28) | (1.28) | |
| Apurimac | | 0.776^***^ | 0.0699^***^ | |
|  | | (4.28) | (4.29) | |
| Arequipa | | 0.974^***^ | 0.0877^***^ | |
|  | | (4.58) | (4.59) | |
| Ayacucho | | 0.771^***^ | 0.0695^***^ | |
|  | | (4.31) | (4.30) | |
| Cajamarca | | 1.133^***^ | 0.102^***^ | |
|  | | (7.58) | (7.75) | |
| Cusco | | -0.532^***^ | -0.0479^***^ | |
|  | | (2.80) | (2.82) | |
| Huancavelica | | 1.766^***^ | 0.159^***^ | |
|  | | (10.27) | (10.49) | |
| Huanuco | | 1.471^***^ | 0.1324^***^ | |
|  | | (8.94) | (9.04) | |
| Ica | | 0.723^**^ | 0.065^***^ | |
|  | | (2.09) | (2.09) | |
| Junin | | -0.074 | -0.00666 | |
|  | | (0.42) | (0.42) | |
| La Libertad | | 0.385^*^ | 0.03468^*^ | |
|  | | (1.75) | (1.75) | |
| Lambayeque | | -0.808^***^ | -0.0728^***^ | |
|  | | (2.59) | (2.59) | |
| Lima | | 2.205^***^ | 0.1985^***^ | |
|  | | (10.22) | (10.43) | |
| Loreto | | 1.912^***^ | 0.1722^***^ | |
|  | | (9.87) | (10.01) | |
| Madre De Dios | | -2.465^***^ | -0.2219^***^ | |
|  | | (7.77) | (7.84) | |
| Moquegua | | 2.484^***^ | 0.2237^***^ | |
|  | | (11.38) | (11.88) | |
| Pasco | | 3.782^***^ | 0.3406^***^ | |
|  | | (20.01) | (22.53) | |
| Piura | | 0.917^***^ | 0.0826^***^ | |
|  | | (4.85) | (4.88) | |
| Puno | | -0.0263 | -0.00237 | |
|  | | (0.14) | (0.14) | |
| San Martin | | -0.198 | -0.0179 | |
|  | | (1.10) | (1.10) | |
| Tacna | | 2.554^***^ | 0.2299^***^ | |
|  | | (12.67) | (13.28) | |
| Tumbes | | 0.876^**^ | 0.07886^**^ | |
|  | | (2.46) | (2.47) | |
| Ucayali | | -2.043^***^ | -0.184^***^ | |
|  | | (6.91) | (7.06) | |
| Constant | | -3.836^***^ | -0.3454^***^ | |
|  | | (8.93) | (9.26) | |
|  | |  |  | |
| Observations | | 9,654 |  | |
| Robust z-statistics in parentheses for coefficients, standard errors for marginal effects. | | |  |  |
| *** p<0.01, ** p<0.05, * p<0.1 | | |  |  |

Notes:i) omitted eco-region coast; ii) omitted department Amazonas
