# Supplementary material for: De-climatizing food security: Lessons from climate change micro-simulations in Peru
Source: PLoS One. 2019 Sep 27;14(9):e0222483. doi: 10.1371/journal.pone.0222483 (PMC6764669; doi:10.1371/journal.pone.0222483)
Supplement: S6 Table — (DOCX) [file pone.0222483.s007.docx]

Table S6. Predicted Climate: Average (2048-50) by geographic domain: CanES ESM

|  | Average rainfall (mm) | | | Maximum temp (C°) | | | Average temp (C°) | | |
| --- | --- | --- | --- | --- | --- | --- | --- | --- | --- |
|  | baseline | projection (2050) | | baseline | projection (2050) | | baseline | projection (2050) | |
| Geographic domain | 2012 | CanEs 4.5 | CanEs 8.5 | 2012 | CanEs 4.5 | CanEs 8.5 | 2012 | CanEs 4.5 | CanEs 8.5 |
| *Coast North* | 449.9 | 778.7 | 797.3 | 30.8 | 32.9 | 33.2 | 23.2 | 25.2 | 25.5 |
| *Coast Center* | 304.5 | 370.5 | 396.8 | 27.2 | 29.3 | 29.7 | 18.9 | 20.9 | 21.3 |
| *Coast South* | 152.8 | 208.7 | 220.9 | 24.6 | 26.5 | 26.9 | 15.2 | 17.1 | 17.5 |
| *Sierra North* | 899.5 | 946.0 | 954.2 | 23.9 | 25.4 | 25.7 | 15.4 | 16.9 | 17.2 |
| *Sierra Center* | 907.0 | 935.1 | 944.6 | 20.4 | 21.6 | 21.9 | 10.6 | 12.1 | 12.4 |
| *Sierra South* | 913.3 | 1052.6 | 1089.3 | 20.2 | 21.5 | 21.8 | 9.5 | 11.0 | 11.3 |
| *Rainforest* | 1617.7 | 1638.7 | 1627.5 | 29.7 | 31.6 | 31.9 | 21.4 | 23.4 | 23.6 |
|  |  |  |  |  |  |  |  |  |  |
| ***Total*** | **995.4** | **1081.4** | **1092.4** | **24.0** | **25.5** | **25.8** | **14.7** | **16.3** | **16.6** |
