# Supplementary material for: De-climatizing food security: Lessons from climate change micro-simulations in Peru
Source: PLoS One. 2019 Sep 27;14(9):e0222483. doi: 10.1371/journal.pone.0222483 (PMC6764669; doi:10.1371/journal.pone.0222483)
Supplement: S7 Table — (DOCX) [file pone.0222483.s008.docx]

Table S7. Predicted Climate: Average (2048-50) by geographic domain: CNR ESM

|  | Average rainfall (mm) | | | Maximum temp (C°) | | | Average temp (C°) | | |
| --- | --- | --- | --- | --- | --- | --- | --- | --- | --- |
| Geographic domain | baseline | projection (2050) | | baseline | projection (2050) | | baseline | projection (2050) | |
|  | 2012 | CNR 4.5 | CNR 8.5 | 2012 | CNR 4.5 | CNR 8.5 | 2012 | CNR 4.5 | CNR 8.5 |
| *Coast North* | 449.9 | 573.0 | 607.8 | 30.8 | 31.7 | 31.9 | 23.2 | 24.1 | 24.4 |
| *Coast Center* | 304.5 | 340.1 | 359.1 | 27.2 | 28.2 | 28.4 | 18.9 | 19.9 | 20.1 |
| *Coast South* | 152.8 | 180.1 | 189.9 | 24.6 | 25.5 | 25.7 | 15.2 | 16.1 | 16.4 |
| *Sierra North* | 899.5 | 885.1 | 893.8 | 23.9 | 24.6 | 24.8 | 15.4 | 16.1 | 16.3 |
| *Sierra Center* | 907.0 | 923.8 | 910.6 | 20.4 | 21.0 | 21.2 | 10.6 | 11.3 | 11.5 |
| *Sierra South* | 913.3 | 916.2 | 913.4 | 20.2 | 20.8 | 21.0 | 9.5 | 10.2 | 10.4 |
| *Rainforest* | 1617.7 | 1689.2 | 1667.8 | 29.7 | 30.4 | 30.6 | 21.4 | 22.2 | 22.4 |
|  |  |  |  |  |  |  |  |  |  |
| ***Total*** | **995.4** | **1027.0** | **1023.1** | **24.0** | **24.6** | **24.8** | **14.7** | **15.4** | **15.6** |
