# Supplementary material for: De-climatizing food security: Lessons from climate change micro-simulations in Peru
Source: PLoS One. 2019 Sep 27;14(9):e0222483. doi: 10.1371/journal.pone.0222483 (PMC6764669; doi:10.1371/journal.pone.0222483)
Supplement: S8 Table — (DOCX) [file pone.0222483.s009.docx]

Table S8. Predicted Climate: Average (2048-50) by geographic domain: MPI ESM

|  | Average rainfall (mm) | | | Maximum temp (C°) | | | Average temp (C°) | | |
| --- | --- | --- | --- | --- | --- | --- | --- | --- | --- |
| Geographic domain | baseline | projection (2050) | | baseline | projection (2050) | | baseline | projection (2050) | |
|  | 2012 | MPI 4.5 | MPI 8.5 | 2012 | MPI 4.5 | MPI 8.5 | 2012 | MPI 4.5 | MPI 8.5 |
| *Coast North* | 449.9 | 621.3 | 726.6 | 30.8 | 32.0 | 32.4 | 23.2 | 24.5 | 24.9 |
| *Coast Center* | 304.5 | 339.5 | 361.7 | 27.2 | 28.6 | 29.0 | 18.9 | 20.2 | 20.6 |
| *Coast South* | 152.8 | 183.6 | 196.6 | 24.6 | 25.9 | 26.4 | 15.2 | 16.5 | 16.9 |
| *Sierra North* | 899.5 | 982.0 | 1019.5 | 23.9 | 24.9 | 25.1 | 15.4 | 16.4 | 16.8 |
| *Sierra Center* | 907.0 | 949.1 | 958.5 | 20.4 | 21.3 | 21.6 | 10.6 | 11.7 | 12.0 |
| *Sierra South* | 913.3 | 945.8 | 959.7 | 20.2 | 21.2 | 21.5 | 9.5 | 10.6 | 10.9 |
| *Rainforest* | 1617.7 | 1690.5 | 1717.1 | 29.7 | 30.7 | 31.0 | 21.4 | 22.5 | 22.9 |
|  |  |  |  |  |  |  |  |  |  |
| ***Total*** | **995.4** | **1060.6** | **1087.5** | **24.0** | **24.9** | **25.2** | **14.7** | **15.8** | **16.1** |
