# Supplementary material for: De-climatizing food security: Lessons from climate change micro-simulations in Peru
Source: PLoS One. 2019 Sep 27;14(9):e0222483. doi: 10.1371/journal.pone.0222483 (PMC6764669; doi:10.1371/journal.pone.0222483)
Supplement: S9 Table — (DOCX) [file pone.0222483.s010.docx]

**Table S9. Effect of CNR climate simulations on yields.**

|  | Yields per ha (Törnqvist index) | | | Prediction with simulated climate variables | | | |
| --- | --- | --- | --- | --- | --- | --- | --- |
| Geographic domain | No. hh | Baseline | Model  Prediction | Prediction CNR 4.5 | diff % | Prediction CNR 8.5 | diff % |
| *Coast North* | 105,954 | 6.645 | 6.586 | 6.644 | 5.8% | 6.615 | 2.9% |
| *Coast Center* | 24,522 | 7.180 | 7.332 | 7.368 | 3.6% | 7.343 | 1.1% |
| *Coast South* | 10,629 | 6.519 | 6.615 | 6.720 | 10.5% | 6.726 | 11.2% |
| *Sierra North* | 327,174 | 5.303 | 5.304 | 5.336 | 3.2% | 5.407 | 10.4% |
| *Sierra Center* | 461,774 | 5.840 | 5.840 | 5.938 | 9.8% | 5.903 | 6.3% |
| *Sierra South* | 488,793 | 5.431 | 5.563 | 5.695 | 13.2% | 5.593 | 3.0% |
| *Rainforest* | 453,161 | 5.179 | 5.150 | 5.015 | -13.5% | 4.967 | -18.3% |
|  |  |  |  |  |  |  |  |
| **Total** | 1,931,197 | **5.566** | **5.593** | **5.638** | **4.5%** | **5.604** | **1.1%** |
