# Supplementary material for: De-climatizing food security: Lessons from climate change micro-simulations in Peru
Source: PLoS One. 2019 Sep 27;14(9):e0222483. doi: 10.1371/journal.pone.0222483 (PMC6764669; doi:10.1371/journal.pone.0222483)
Supplement: S11 Table — (DOCX) [file pone.0222483.s012.docx]

**Table S11. Effect of climate simulations on mean caloric consumption: CanES Model.**

|  | Kcal/person/day | | Prediction with simulated climate variables | | | |
| --- | --- | --- | --- | --- | --- | --- |
| Geographic domain | Baseline | Model  Prediction | Prediction CANES 4.5 | diff % | Prediction CANES 8.5 | diff % |
| *Coast North* | 2,650 | 2,572 | 2,584 | 0.421% | 2,592 | 0.679% |
| *Coast Center* | 2,836 | 2,829 | 2,858 | 0.688% | 2,890 | 1.369% |
| *Coast South* | 3,077 | 2,943 | 3,003 | 1.389% | 3,031 | 2.019% |
| *Sierra North* | 2,271 | 2,118 | 2,122 | 0.198% | 2,117 | -0.016% |
| *Sierra Center* | 2,423 | 2,211 | 2,213 | 0.078% | 2,208 | -0.116% |
| *Sierra South* | 2,557 | 2,398 | 2,399 | 0.058% | 2,389 | -0.332% |
| *Rainforest* | 2,628 | 2,387 | 2,370 | -0.475% | 2,375 | -0.333% |
|  |  |  |  |  |  |  |
| ***Total*** | **2,503** | **2,323** | **2,323** | **0.029%** | **2,321** | **-0.073%** |
