# Supplementary material for: De-climatizing food security: Lessons from climate change micro-simulations in Peru
Source: PLoS One. 2019 Sep 27;14(9):e0222483. doi: 10.1371/journal.pone.0222483 (PMC6764669; doi:10.1371/journal.pone.0222483)
Supplement: S12 Table — (DOCX) [file pone.0222483.s013.docx]

**Table S12. Effect of climate simulations on mean caloric consumption: CNR Model.**

|  | Kcal/person/day | | Prediction with simulated climate variables | | | |
| --- | --- | --- | --- | --- | --- | --- |
| Geographic domain | Baseline | Model  Prediction | Prediction CNR 4.5 | diff % | Prediction CNR 8.5 | diff % |
| *Coast North* | 2,650 | 2,572 | 2,580 | 0.278% | 2,577 | 0.159% |
| *Coast Center* | 2,836 | 2,829 | 2,838 | 0.243% | 2,831 | 0.088% |
| *Coast South* | 3,077 | 2,943 | 2,978 | 0.833% | 2,980 | 0.863% |
| *Sierra North* | 2,271 | 2,118 | 2,119 | 0.062% | 2,122 | 0.164% |
| *Sierra Center* | 2,423 | 2,211 | 2,216 | 0.219% | 2,214 | 0.146% |
| *Sierra South* | 2,557 | 2,398 | 2,407 | 0.360% | 2,401 | 0.111% |
| *Rainforest* | 2,628 | 2,387 | 2,381 | -0.275% | 2,366 | -0.677% |
|  |  |  |  |  |  |  |
| ***Total*** | **2,503** | **2,323** | **2,326** | **0.123%** | **2,321** | **-0.033%** |
