# Supplementary material for: De-climatizing food security: Lessons from climate change micro-simulations in Peru
Source: PLoS One. 2019 Sep 27;14(9):e0222483. doi: 10.1371/journal.pone.0222483 (PMC6764669; doi:10.1371/journal.pone.0222483)
Supplement: S13 Table — (DOCX) [file pone.0222483.s014.docx]

**Table S13. Effect of climate simulations on mean caloric consumption: MPI Model.**

|  | Kcal/person/day | | Prediction with simulated climate variables | | | |
| --- | --- | --- | --- | --- | --- | --- |
| Geographic domain | Baseline | Model  Prediction | Prediction MPI 4.5 | diff % | Prediction MPI 8.5 | diff % |
| *Coast North* | 2,650 | 2,572 | 2,587 | 0.534% | 2,579 | 0.251% |
| *Coast Center* | 2,836 | 2,829 | 2,858 | 0.691% | 2,843 | 0.356% |
| *Coast South* | 3,077 | 2,943 | 3,016 | 1.674% | 2,997 | 1.252% |
| *Sierra North* | 2,271 | 2,118 | 2,120 | 0.085% | 2,122 | 0.184% |
| *Sierra Center* | 2,423 | 2,211 | 2,214 | 0.113% | 2,213 | 0.103% |
| *Sierra South* | 2,557 | 2,398 | 2,405 | 0.282% | 2,400 | 0.108% |
| *Rainforest* | 2,628 | 2,387 | 2,371 | -0.555% | 2,362 | -0.800% |
|  |  |  |  |  |  |  |
| ***Total*** | 2,503 | 2,323 | 2,325 | 0.059% | 2,321 | -0.050% |
