# Supplementary material for: De-climatizing food security: Lessons from climate change micro-simulations in Peru
Source: PLoS One. 2019 Sep 27;14(9):e0222483. doi: 10.1371/journal.pone.0222483 (PMC6764669; doi:10.1371/journal.pone.0222483)
Supplement: S14 Table — (DOCX) [file pone.0222483.s015.docx]

Table S14. Effect of climate simulations on vulnerability: CNR Model.

|  |  |  | Vulnerability (Probability) | | | |
| --- | --- | --- | --- | --- | --- | --- |
| Geographic domain | Obs. | Baseline | Prediction CNR 4.5 | diff % | Prediction CNR 8.5 | diff % |
| *Coast North* | 577,462 | 0.2391 | 0.2375 | -0.67% | 0.2381 | -0.42% |
| *Coast Center* | 201,227 | 0.2330 | 0.2321 | -0.39% | 0.2325 | -0.21% |
| *Coast South* | 69,564 | 0.2485 | 0.2461 | -0.97% | 0.2460 | -1.01% |
| *Sierra North* | 1,435,993 | 0.4055 | 0.4050 | -0.12% | 0.4043 | -0.30% |
| *Sierra Center* | 2,024,206 | 0.3781 | 0.3765 | -0.42% | 0.3770 | -0.29% |
| *Sierra South* | 1,664,126 | 0.3087 | 0.3065 | -0.71% | 0.3080 | -0.23% |
| *Rainforest* | 1,677,269 | 0.3176 | 0.3196 | 0.63% | 0.3214 | 1.20% |
|  |  |  |  |  |  |  |
| ***Total*** | **7,649,847** | 0.3394 | 0.3387 | -0.21% | 0.3395 | 0.03% |
