# Supplementary material for: De-climatizing food security: Lessons from climate change micro-simulations in Peru
Source: PLoS One. 2019 Sep 27;14(9):e0222483. doi: 10.1371/journal.pone.0222483 (PMC6764669; doi:10.1371/journal.pone.0222483)
Supplement: S15 Table — (DOCX) [file pone.0222483.s016.docx]

Table S15. Effect of climate simulations on vulnerability: MPI Model.

|  |  |  | Vulnerability (Probability) | | | |
| --- | --- | --- | --- | --- | --- | --- |
| Geographic domain | Obs. | Baseline | Prediction MPI 4.5 | diff % | Prediction MPI 8.5 | diff % |
| *Coast North* | 577,462 | 0.2391 | 0.2361 | -1.25% | 0.2377 | -0.59% |
| *Coast Center* | 201,227 | 0.2330 | 0.2312 | -0.77% | 0.2319 | -0.47% |
| *Coast South* | 69,564 | 0.2485 | 0.2441 | -1.77% | 0.2450 | -1.41% |
| *Sierra North* | 1,435,993 | 0.4055 | 0.4049 | -0.15% | 0.4042 | -0.32% |
| *Sierra Center* | 2,024,206 | 0.3781 | 0.3772 | -0.24% | 0.3773 | -0.21% |
| *Sierra South* | 1,664,126 | 0.3087 | 0.3070 | -0.55% | 0.3080 | -0.23% |
| *Rainforest* | 1,677,269 | 0.3176 | 0.3208 | 1.01% | 0.3221 | 1.42% |
|  |  |  |  |  |  |  |
| ***Total*** | **7,649,847** | 0.3394 | 0.3391 | -0.09% | 0.3396 | 0.06% |
